# miR-1260b, mediated by YY1, activates KIT signaling by targeting SOCS6 to regulate cell proliferation and apoptosis in NSCLC

**DOI:** 10.1038/s41419-019-1390-y

**Published:** 2019-02-08

**Authors:** Yang Xia, Ke Wei, Feng-Ming Yang, Liu-Qing Hu, Chun-Feng Pan, Xiang-Long Pan, Wei-Bing Wu, Jun Wang, Wei Wen, Zhi-Cheng He, Jing Xu, Xin-Feng Xu, Quan Zhu, Liang Chen

**Affiliations:** 1 Department of Thoracic Surgery, the First Affiliated Hospital of Nanjing Medical University, Nanjing, 210029 China; 2 Department of Oncology, the First Affiliated Hospital of Nanjing Medical University, Nanjing, 210029 China; 3 Department of Anesthesiology, the First Affiliated Hospital of Nanjing Medical University, Nanjing, 210029 China

## Abstract

Non-small cell lung cancer (NSCLC) is one of the most common aggressive malignancies. miRNAs have been identified as important biomarkers and regulators of NSCLC. However, the functional contributions of miR-1260b to NSCLC cell proliferation and apoptosis have not been studied. In this study, miR-1260b was upregulated in NSCLC plasma, tissues, and cell lines, and its high expression was correlated with tumor size and progression. Functionally, miR-1260b overexpression promoted cell proliferation and cell cycle, conversely inhibited cell apoptosis and senescence. Mechanically, miR-1260b negatively regulated SOCS6 by directly binding to its 3′-UTR. Furthermore, miR-1260b-mediated suppression of SOCS6 activated KIT signaling. Moreover, YY1 was an upstream regulator of miR-1260b. This study is the first to illustrate that miR-1260b, mediated by YY1, activates KIT signaling by targeting SOCS6 to regulate NSCLC cell proliferation and apoptosis, and is a potential biomarker and therapeutic target for NSCLC. In sum, our work provides new insights into the molecular mechanisms of NSCLC involved in cell proliferation and apoptosis.

## Introduction

Lung cancer is one of the most common malignancies in the world^1^. According to statistics from the International Agency for Research on Cancer, there were 1.825 million new cases and 1.59 million cases of lung cancer deaths in 2012, accounting for 13.0% and 19.4% of the incidence and mortality of all cancers^2^. In China, there were 733,000 lung cancer cases and 61,000 lung cancer deaths in 2015, which ranks first in the incidence and death of all malignant tumors^3^. Lung cancer has become a major public health problem that threatens the lives and health of people in our country and even the world. Of them, 80% of lung cancers are mainly non-small cell lung cancer (NSCLC). Although the traditional treatment methods such as surgical resection, radiotherapy and chemotherapy, and targeted therapy have been continuously improved, the overall 5-year survival rate is still poor^1^. One of the main factors affecting its therapeutic effect and prognosis is the abnormal proliferation of tumor cells. Therefore, exploring the molecular mechanisms of cell proliferation, finding the new molecular targets, so as to improve the treatment effect is a hotpot in the current research field of lung cancer.

Cancer is a type of disease that involves multiple genetic alterations, resulting in the continued proliferation of cells. In the development of tumors, changes in various regulatory factors, including the activation of oncogenes and the inactivation of tumor suppressor genes, are currently important anti-tumor targets. Abnormal expression of Cyclins family is one type of the important regulatory factors of tumor cell proliferation^4^. Meanwhile, the genes that participate in the regulation of cell apoptosis are apoptosis-activated genes (Caspases family) and apoptosis-suppressing genes (Bcl-2 family)^5^. Recent studies report that p21 is associated with cell senescence, and induces spontaneous cell death^6^.

miRNAs, noncoding RNAs, regulate the target genes by inhibiting mRNA translation or enhancing mRNA degradation^7,8^. Recently, emerging studies have reported that miRNAs are involved in cell proliferation, growth, apoptosis, and differentiation^9,10^. In lung cancer, miR-19b promotes proliferation and apoptosis resistance by modulating PP2A and BIM^11^. miR-218 suppresses lung cancer progression by regulating IL-6/STAT3 signaling pathway^12^. More studies are needed to explore the roles of miRNAs in lung cancer diagnosis and development, so as to reduce the mortality of lung cancer patients.

Our current study illustrated that miR-1260b played a tumor-promoting role in human NSCLC. We reported herein the elucidation of a novel pathway in NSCLC, in which YY1-regulated miR-1260b targets SOCS6 and thereby enhances the KIT signaling.

## Results

### miR-1260b was increased in the plasma of NSCLC patients

At first, the data of gene expression microarray (GSE68951)^13^ were downloaded from Gene Expression Omnibus (GEO) database and the peripheral blood profiles of patients with NSCLC and controls were obtained (Additional file 1: Figure. S1a–c). Of them, the role of miR-1260b overexpression in NSCLC was rarely reported. Next, we detected miR-1260b expression in the plasma of NSCLC patients (*n* = 90) and controls (*n* = 30). The result showed higher-expression of miR-1260b in the plasma of NSCLC patients as compared with the controls (Additional file 1: Figure. S1d). All the data revealed that miR-1260b might serve as a novel biomarker of NSCLC.

### miR-1260b was also upregulation both in NSCLC tissues and cell lines

**Fig. 1:**
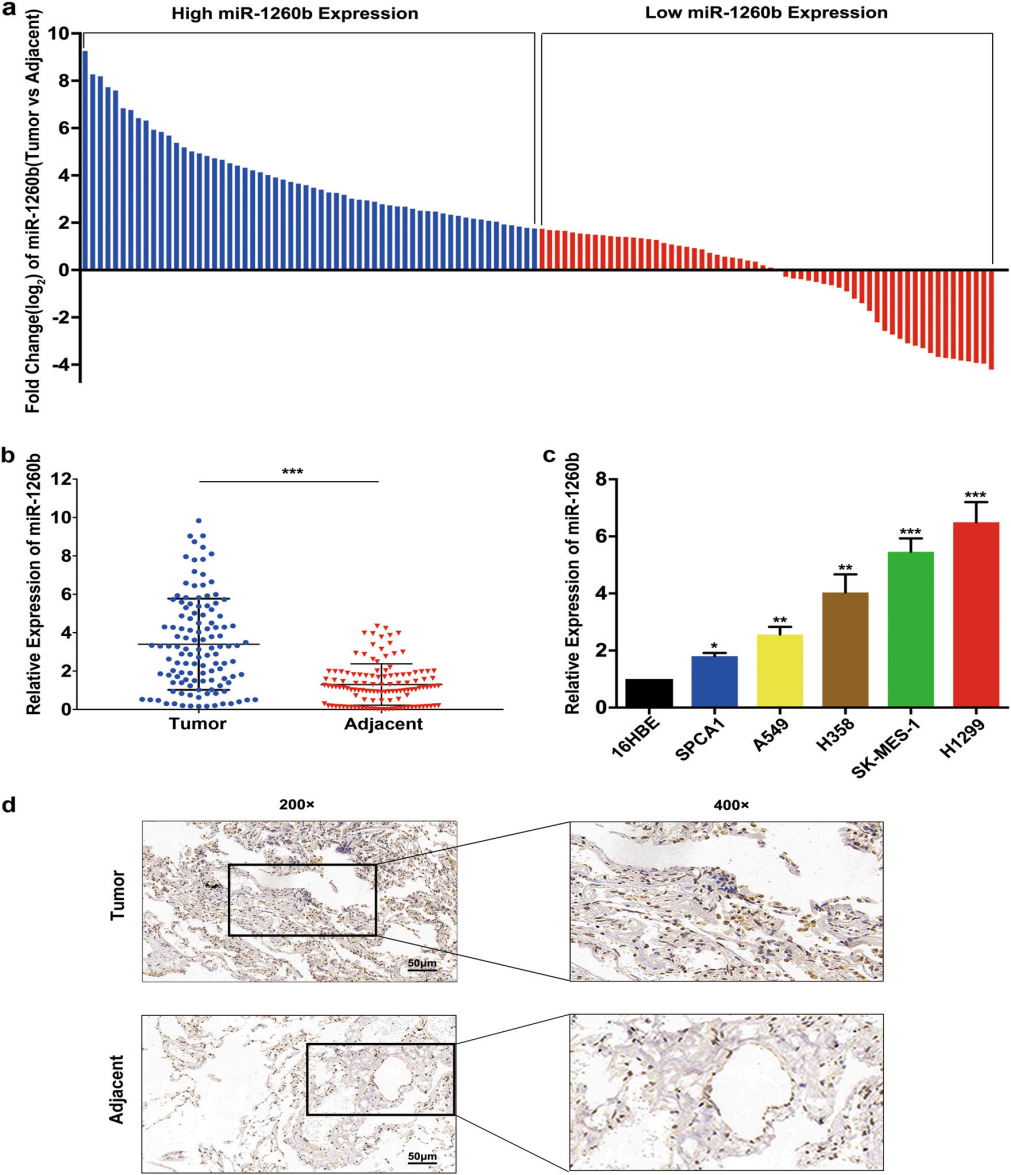
miR-1260b was upregulation both in NSCLC tissues and cell lines. **a** The fold change (log2 change) in miR-1260b level between 120 pairs of NSCLC and adjacent tissues was evaluated, and were divided into a high miR-1260b expression group (*n* = 60) and a low miR-1260b expression group (*n* = 60) based on the median miR-1260b level. **b** miR-1260b level in 120 NSCLC tissues and paired adjacent tissues was investigated by qRT-PCR. **c** miR-1260b level in normal lung 16HBE epithelial cells and five NSCLC cell lines was investigated by qRT-PCR; U6 was used as an internal control. **d** Representative images of in situ hybridization for miR-1260b in the tissues and adjacent tissues. Magnification: × 200 and × .400. The data are shown as the mean ± SD (*P < 0.05; **P < 0.01)

Next, we detected the level of miR-1260b in NSCLC tissues and adjacent tissues (*n* = 120). According to the median, the samples were divided into high and low expression groups (Fig. 1a). The level of miR-1260b was obviously upregulated in NSCLC tissues, indicating that miR-1260b might contribute into the development of NSCLC (Fig. 1b). As presented in Table 1, miR-1260b was significantly related to tumor size, but no difference was observed among age, gender, tumor type, and smoke. Furthermore, we also uncovered that tumor cell lines had higher miR-1260b expression than 16HBE cells. Of them, H1299 cells had the highest miR-1260b expression, whereas SPCA1 cells had the lowest miR-1260b expression (Fig. 1c). Assessment of miR-1260b expression in an “in-house” localized tumor cohort by in situ hybridization ISH demonstrated that it was expressed primarily in malignant epithelial cells (Fig. 1d). The above findings indicated that miR-1260b might act as an oncogene that participating in NSCLC development.

**Table 1:** Correlation between miR-1260b expression and clinical features in patients with NSCLC

Feature	No.	miR-1260b expression		*P* value
		High	Low	
Age (years)				0.187
≤ 60	45	19	26	
å 60	75	41	34	
Gender				0.356
Male	69	32	37	
Female	51	28	23	
Tumor type				0.157
AC	98	46	52	
SC	22	14	8	
Smoke				0.273
Yes	62	34	28	
No	58	26	32	
Tumor size				0.024*
T1–T2	101	46	55	
T3– T4	19	14	5	

AC: adenocarcinoma; SC: squamous cell carcinoma. *P < 0.05

The tumor size was classified by UICC (The Union for International Cancer Control) version 8

### miR-1260b regulated cell proliferation, cell cycle, apoptosis, and senescence

**Fig. 2:**
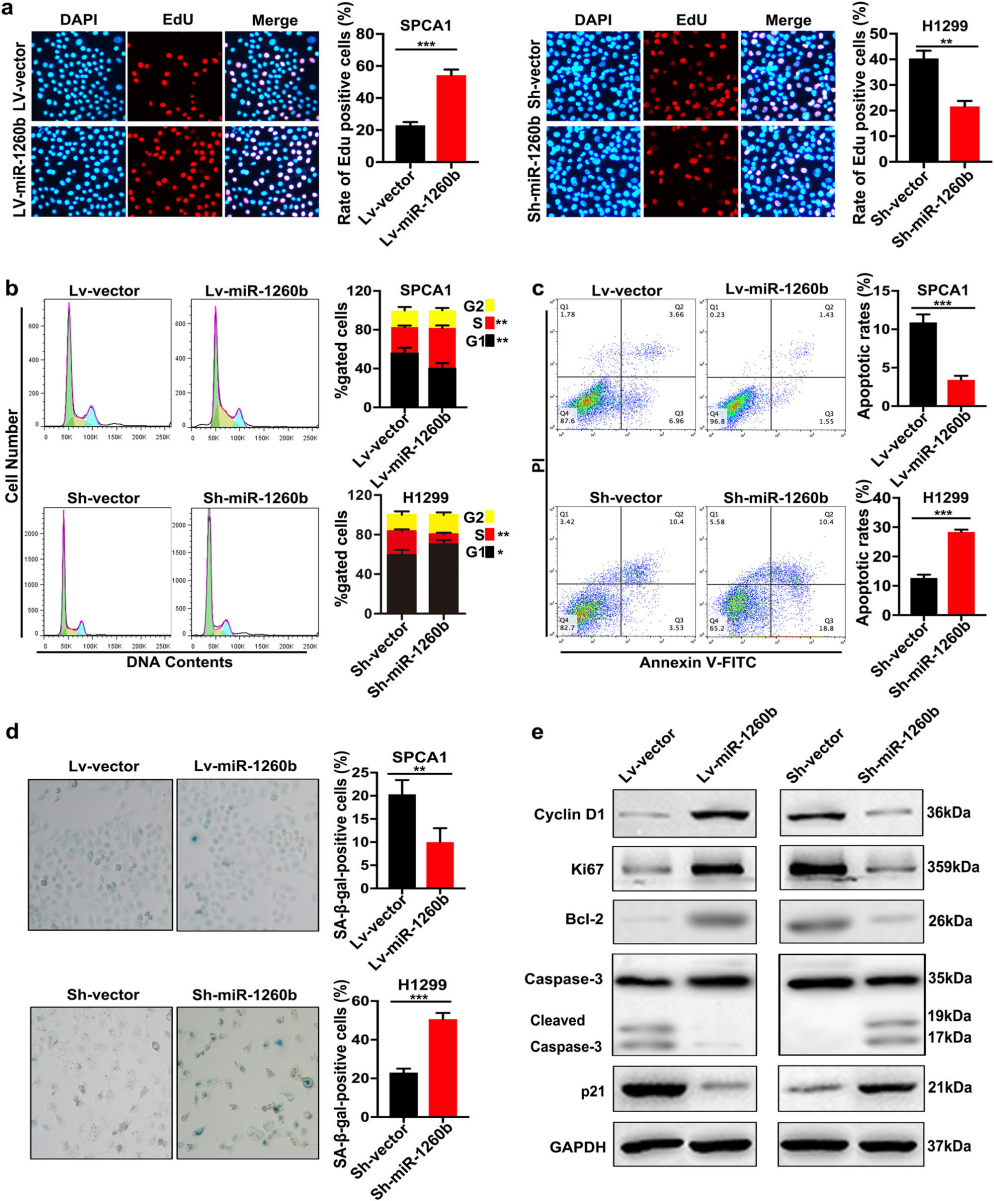
miR-1260b regulated cell proliferation, cell cycle, apoptosis, and senescence. **a** Representative profile of EdU cell growth in SPCA1 cells and H1299 cells after transfection with Lv-miR-1260b and Sh-miR-1260b respectively compared with the control. **b** Effects of miR-1260b alteration on cell cycle distribution of cells. **c** FACS analysis of the effect of miR-1260b expression alteration on cell apoptosis. **d** SA-β-gal staining analysis of the effect of miR-1260b expression alteration on cell senescence. **e** Expression levels of Cyclin D1, Ki67, Bcl-2, Caspase-3, and p21 were detected by western blot. GAPDH was used as an internal control. All experiments are performed three times independently. The data expressed as the mean ± SD (*P < 0.05; **P < 0.01; ***P < 0.001)

To investigate the underlying functions of miR-1260b, gain- and loss-of-function analyses were performed. miR-1260b was upregulated in the Lv-miR-1260b-transfected SPCA1 cells compared with the Lv-vector-transfected cells, and downregulated in the Sh-miR-1260b-transfected H1299 cells relative to the Sh-vector-transfected cells (Additional file 2: Figure. S2a). Both Cell Counting Kit-8 (CCK-8) (Additional file 2: Figure. S2b) and 5-Ethynyl-2’-deoxyuridine (EdU) (Fig. 2a) assays exhibited that upregulation of miR-1260b in SPCA1 cells exerted a promoting effect on cell proliferation, whereas low expression of miR-1260b in H1299 cells exerted an inhibition effect. Examination of flow cytometry showed that cell distribution of S phase in miR-1260b overexpression group was increased in comparison with the control group; miR-1260b low expression caused a G0/G1 cell cycle arrest (Fig. 2b). Moreover, the flow cytometry assay also exhibited that upregulation of miR-1260b in SPCA1 cells exerted an inhibition effect on cell apoptosis, whereas low expression of miR-1260b in H1299 cells exerted a promoting effect (Fig. 2c). To investigate the effect of miR-1260b on cell senescence, detection of SA-β-gal staining was performed. The results revealed that overexpression of miR-1260b in SPCA1 cells exerted an inhibition effect on cell senescence, whereas low expression of miR-1260b in H1299 cells exerted a promoting effect (Fig. 2d). Furthermore, analysis of western blot as presented in Fig. 2e, upregulation of miR-1260b markedly increased Cyclin D1, Ki67, Bcl-2, and decreased cleaved Caspase-3, p21. Knockdown of miR-1260b markedly decreased Cyclin D1, Ki67, Bcl-2 and increased cleaved Caspase-3, p21.

### miR-1260b negatively regulated SOCS6 by directly binding to its 3′-UTR

**Fig. 3:**
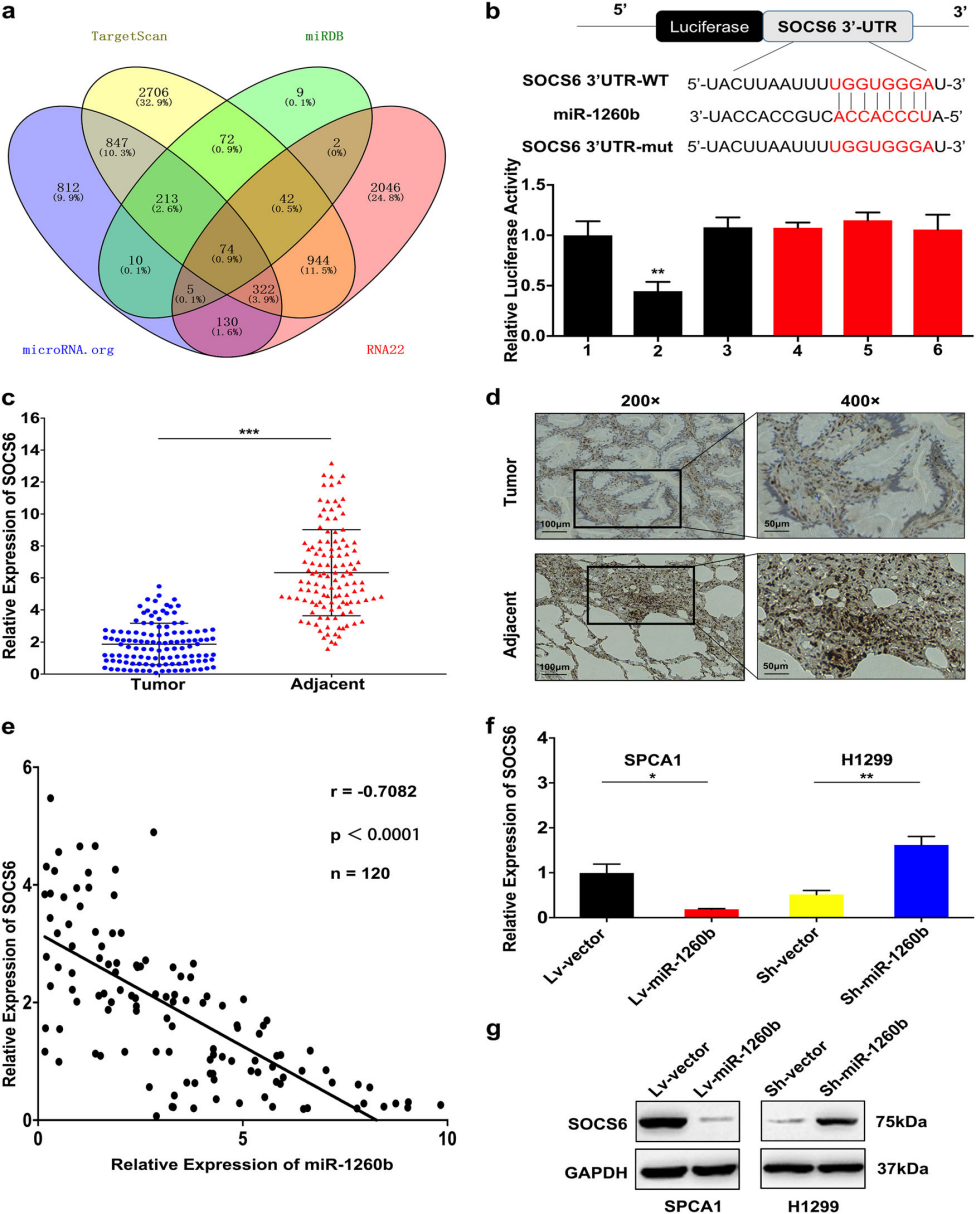
miR-1260b negatively regulated SOCS6 by directly binding to its 3′-UTR. **a** The candidate gene targets were predicted by intersecting outputs from four distinct prediction algorithms (TargetScan, miRDB, RNA22, and microRNA.org). **b** The potential miR-1260b seed region at the 3′-UTR of SOCS6 mRNA was computationally predicted. SPCA1 cells were co-transfected with miR-1260b mimics (or NC) with pGL3-SOCS6 (or pGL3-SOCS6-mut) vector. Luciferase activity was normalized by the ratio of firefly and Renilla luciferase signals. 1: pGL3-SOCS6; 2: pGL3-SOCS6 + miR-1260b mimics; 3: pGL3-SOCS6 + NC; 4: pGL3-SOCS6 mut; 5: pGL3-SOCS6 mut + miR-1260b mimics; 6: pGL3-SOCS6 mut + NC. **c** SOCS6 mRNA in 120 NSCLC tissues and paired adjacent tissues was investigated by qRT-PCR. **d** IHC staining against SOCS6 assay was used to detect the effects of miR-1260b expression alteration on cell proliferation in the tissue samples. Magnification: × 200 and × 400. Scale bar is 100 μm and 50 μm. **e** There was an inverse correlation between miR-1260b and SOCS6. **f**, **g** A negative regulatory effect of miR-1260b on SOCS6 was tested by qRT-PCR and western blot. GAPDH was used as an internal control. The data expressed as the mean ± SD (*P < 0.05; **P < 0.01; ***P < 0.001)

To investigate the potential target of miR-1260b, we predicted candidate gene targets by intersecting outputs from four distinct prediction algorithms, (TargetScan, miRDB, RNA22, and microRNA.org) (Fig. 3a). From the resultant list of 74 genes (Additional file 3: Figure. S3), we focused on the factors that are downregulated in NSCLC. Of them, the bioinformatic software analysis suggested 3′-UTR of SOCS6 binds to miR-1260b with the high score. Thus, a luciferase reporter experiment was conducted to confirm whether 3′-UTR of SOCS6 was a direct target of miR-1260b. The vector containing the site-mutated sequence was not influenced by miR-1260b. However, the relative luciferase intensity of cells with miR-1260b and SOCS6 3′-UTR plasmids was obviously reduced (Fig. 3b). Furthermore, downregulated SOCS6 caused a promotion effect of cell proliferation and an inhibition effect of cell apoptosis (Additional file 4: Figure. S4). In addition, we found decreased SOCS6 expression in the tumor tissues (Fig. 3c, d). There was an inverse correlation between miR-1260b and SOCS6 (*r* = −0.7082; *P* < 0.0001; Fig. 3e). And, to assess SOCS6 expression responses to the changes of miR-1260b, both the results of qRT-PCR (Fig. 3f) and western blot (Fig. 3g) showed that a negative regulatory effect of miR-1260b on SOCS6. Upregulated miR-1260b resulted in decreased SOCS6 expression; meanwhile, knockdown miR-1260b resulted in increased SOCS6 expression. In summary, all the data revealed that miR-1260b could influence SOCS6 expression via directly binding its 3′-UTR.

### Reconstitution or knockdown of SOCS6 partially rescued the miR-1260b-mediated effects

To confirm whether the regulatory effect of miR-1260b was depending on SOCS6, we conducted a rescue experiment. SPCA1 cells were co-transfected with Lv-miR-1260b and Lv-SOCS6. H1299 cells were co-transfected with Sh-miR-1260b and si-SOCS6. The efficiency of transfection was confirmed (Additional file 5: Figure. S5a). Both CCK-8 and EdU assays indicated that upregulation of SOCS6 partially abolished the enhancement of SPCA1 cell proliferation induced by up-regulating miR-1260b. The rescue effects were observed in H1299 transfected with Sh-miR-1260b + si-SOCS6 compared with the control (Additional file 5: Figure. S5b, c). Furthermore, in SPCA1 transfected with Lv-miR-1260b + Lv-SOCS6 compared with the control, cell cycle, cell apoptosis, and senescence indeed reversed to some extent, and decreased Cyclin D1, Ki67, Bcl-2, and increased cleaved Caspase-3, p21 were observed. The rescue effects were also observed in H1299 transfected with Sh-miR-1260b + si-SOCS6 compared with the control (Additional file 6: Figure. S6a–d).

### miR-1260b promoted xenograft tumor formation

**Fig. 4:**
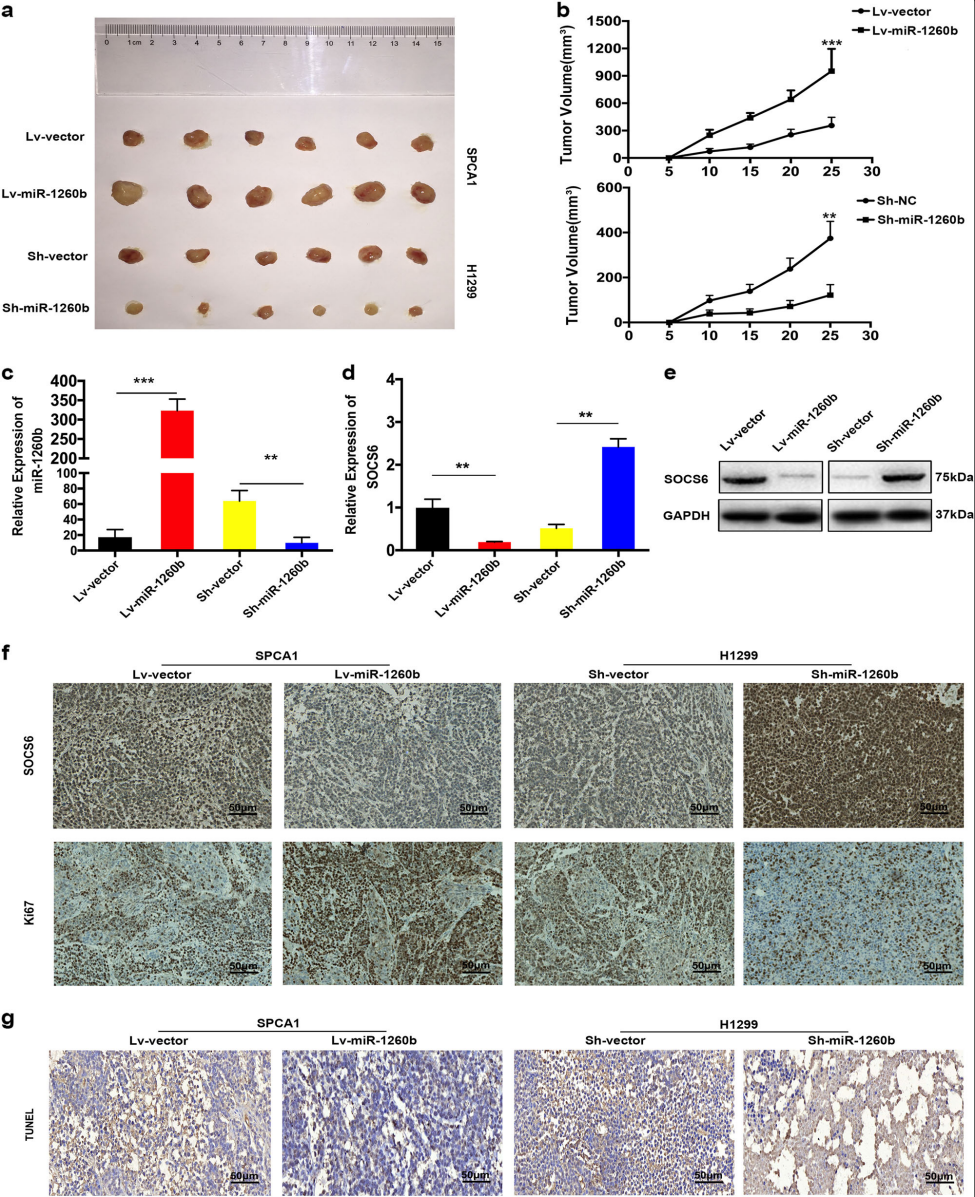
miR-1260b promoted xenograft tumor formation. **a** Photographs of tumors obtained from the different groups of nude mice transfected with Lv-miR-1260b and Sh-miR-1260b, respectively. **b** Growth curve of tumor volumes were calculated. **c–e** The levels of miR-1260b and SOCS6 in the implanted tumors were detected by miRNA RT–PCR and western blot. GAPDH was used as an internal control. **f** SOCS6 and Ki67 expression levels the samples collected from nude mice were analyzed by IHC. Scale bar is 50 μm. **g** TUNEL assay were used to evaluate the effect of miR-1260b expression alteration on cell apoptosis in the samples collected from nude mice. Scale bar is 50 μm. The data expressed as the mean ± SD (*P < 0.05; **P < 0.01; ***P < 0.001)

In vivo, the transfected cells were orthotopically inoculated into each nude mice as well as scramble in the left flank. The mice inoculated with miR-1260b overexpression SPCA1 cells had increased tumor volume (Fig. 4a, b). As expected, the tumor volume of Sh-miR-1260b group was decreased relative to those of Sh-vector group. Furthermore, increased miR-1260b and decreased SOCS6 was dramatically emerged in miR-1260b overexpressing group; meanwhile, the inverse effect was observed in Sh-miR-1260b group (Fig. 4c–f). IHC staining against Ki67 were consistent with the observation that miR-1260b promoted NSCLC proliferation (Fig. 4f). The analysis of terminal deoxynucleotidyl transferase dUTP nick end labeling (TUNEL) exhibited that upregulated miR-1260b in SPCA1 cells presented an inhibition effect on cell apoptosis, whereas low expression of miR-1260b in H1299 cells exerted a promoting effect on cell apoptosis (Fig. 4g). Conclusion, these findings demonstrated that miR-1260b promoted tumor formation in vivo.

### miR-1260b-mediated suppression of SOCS6 activated KIT signaling

**Fig. 5:**
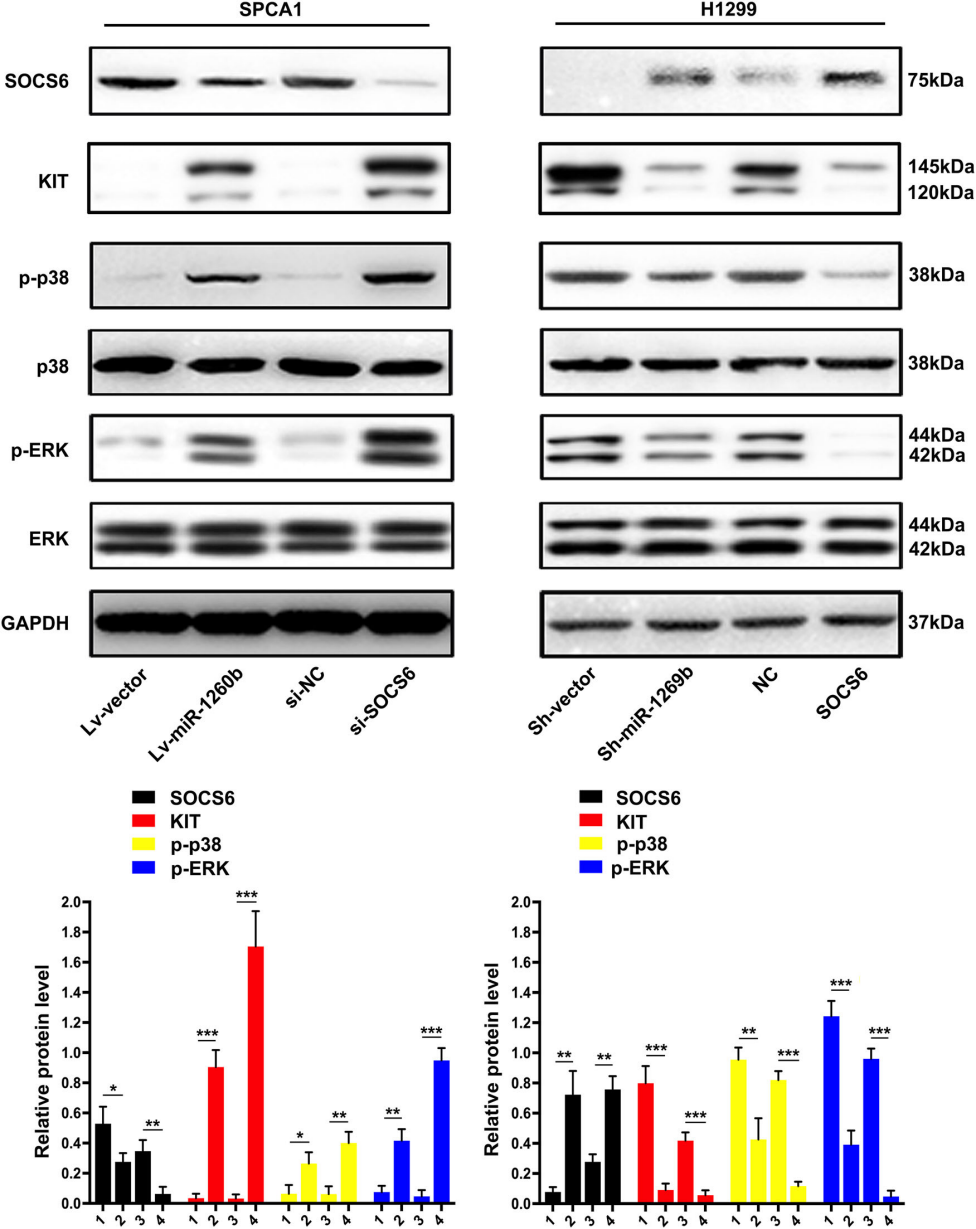
miR-1260b-mediated suppression of SOCS6 activated KIT signaling. Expression levels of SOCS6, KIT, p-p38, p-ERK, and ERK were detected by western blot. GAPDH was used as an internal control. 1: Lv-vector, 2: Lv-miR-1260b, 3: si-NC, 4: si-SOCS6 in SPCA1 cell line; 1: Sh-vector, 2: Sh-miR-1260b, 3: NC, 4: SOCS6 in H1299 cell line. The data expressed as the mean ± SD (*P < 0.05; **P < 0.01; ***P < 0.001)

SOCS6 gene is able to interacting with KIT through the stimulation of the KIT ligand stem cell factor^14^. The major SOCS6-binding site in KIT is pY567, which selectively involved the SH2 domain of SOCS6^15^. This interactive function is association with the negative regulatory effect of the different signaling, such as phosphorylation of ERK1/2 and p38, so as to inversely influence cell proliferative ability^14^. Otherwise, the influence mediated by SOCS6 is owing to the ubiquitination-directed degradation of KIT^15^. Thus, we speculated that enhanced activity of KIT-regulated pathways in response to loss of SOCS6 might be a mechanism by which miR-1260b promotes cell proliferation. Indeed, KIT expression was elevated in response to miR-1260b overexpression and SOCS6 knockdown. Moreover, miR-1260b and si-SOCS6 both enhanced the phosphorylation of p38 and ERK (Fig. 5).

### YY1 was an upstream regulator of miR-1260b

**Fig. 6:**
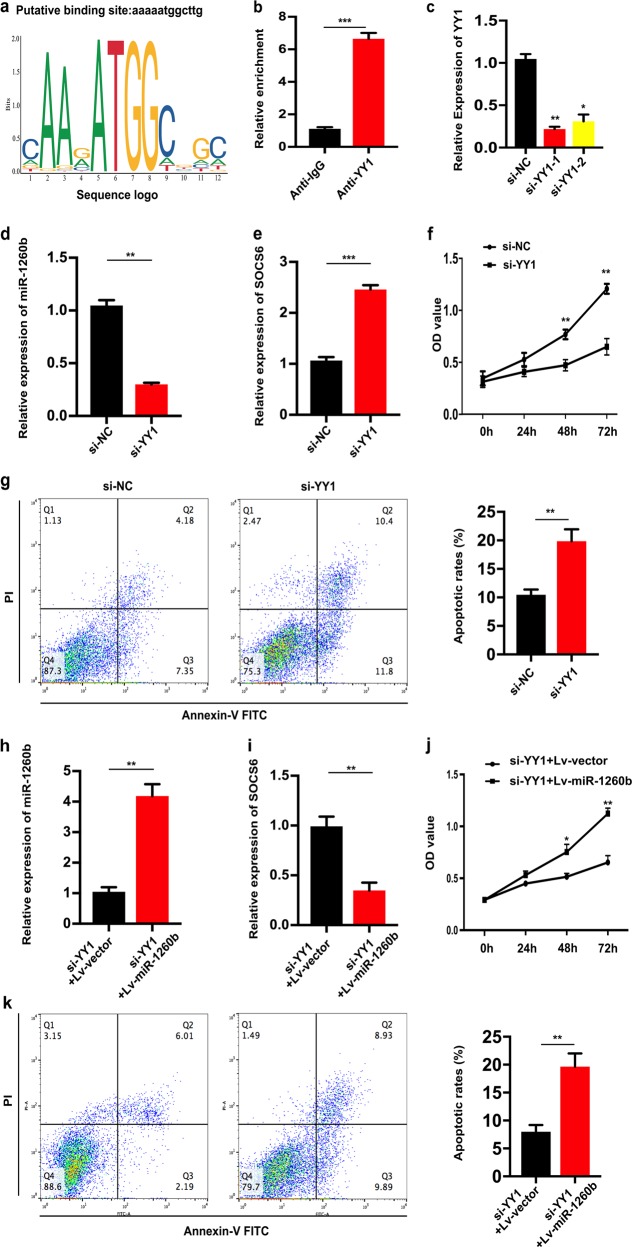
YY1 was an upstream regulator of miR-1260b. **a** Based on JASPAR database, the putative score of YY1 binding to miR-1260b was the highest. **b** ChIP assay indicated the YY1 binds to the putative binding site upstream of miR-1260b. **c** The effect of knockdown YY1 by siRNA was identified by qRT-PCR. **d**, **e** Levels of miR-1260b and SOCS6 responding downregulated YY1 were detected by qRT-PCR. **f**, **g** CCK-8 and flow cytometric assays were the influence of downregulated YY1 on cell proliferation and apoptosis respectively. **h**, **i** The relative expression level of miR-1260b or SOCS6 was accessed by qRT-PCR after co-transfection si-YY1 with Lv-miR-1260b or Lv-vector. **j**, **k** Cell proliferation and cell apoptosis was determined after co-transfected with si-YY1 and Lv-miR-1260b or Lv-vector. The data expressed as the mean ± SD (*P < 0.05; **P < 0.01; ***P < 0.001)

To identify upstream regulators that may be responsible for miR-1260b overexpression in NSCLC, based on JASPAR database, we found that 33 genes might be potential upstream regulator of miR-1260b. Of them, the putative score of YY1 binding to miR-1260b was the highest (Fig. 6a). YY1 is upregulated in lung cancer tissues, and is related to tumor size. Besides, YY1 acts as a multifunctional transcription factor, and is able to regulate NSCLC cell proliferation and apoptosis^16^. Furthermore, ChIP assay indicated that YY1 binds to the putative binding site upstream of miR-1260b (Fig. 6b). The effect of knockdown YY1 by small interfering RNA (siRNA) was identified (Fig. 6c). Downregulation of YY1 led to decreased miR-1260b and increased SOCS6 expression, which indicated that there was an axis among YY1/miR-1260b/SOCS6 signaling (Fig. 6d, e). Downregulated YY1 could repress cell proliferative ability and promote apoptosis (Fig. 6f, g). Subsequently, we recruited a rescue assay to figure out whether miR-1260b could rescue the si-YY1 effect. As shown in Fig. 6h, i, after co-transfection of Lv-miR-1260b or Lv-vector with si-YY1, the expression level of miR-1260b and SOCS6 were detected. Moreover, the results showed that miR-1260b overexpression could partially rescue the effects of si-YY1 on cell proliferation and apoptosis in NSCLC cell lines (Fig. 6j, k). Overall, the total data indicated that YY1 contributed to the promotion of cell proliferation and inhibition of cell apoptosis by modulating miR-1260b transcription.

## Discussion

**Fig. 7:**
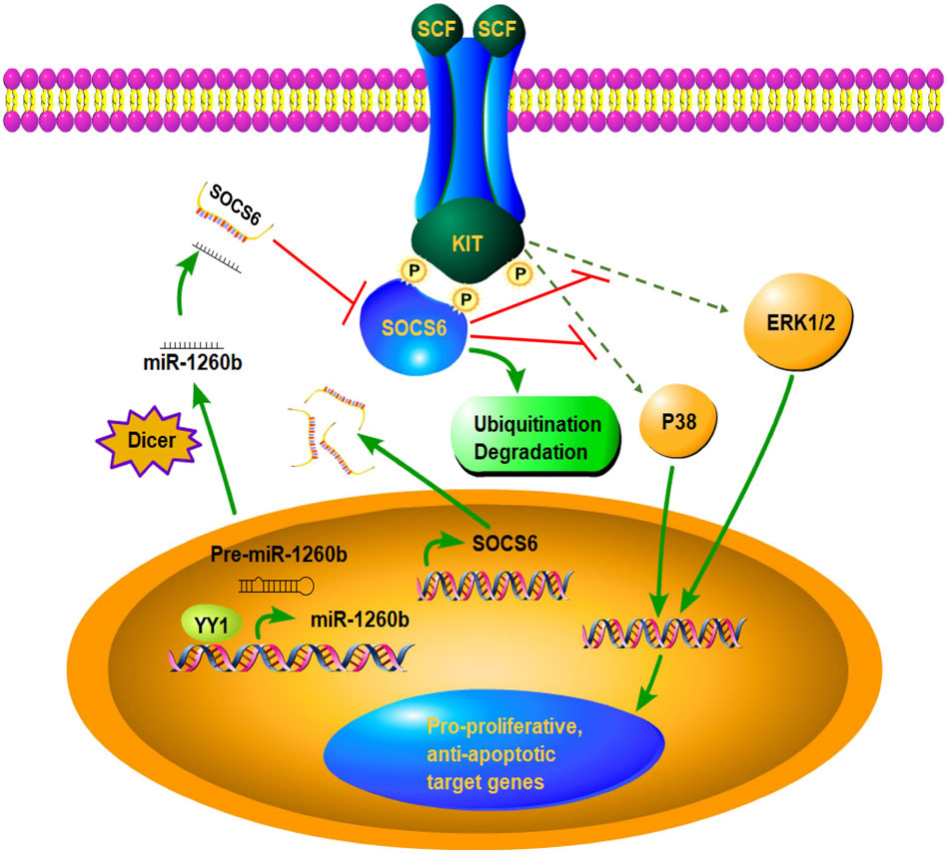
**Schematic diagram for miR-1260b, mediated by YY1, activates KIT signaling by targeting SOCS6 to regulate cell proliferation and apoptosis in NSCLC.** YY1 was verified to bind to miR-1260b promoter and activates miR-1260b expression in NSCLC. miR-1260b regulate cell proliferation and apoptosis in NSCLC by targeting SOCS6 and thereby activates KIT sgnaling

In this study, we have provided new insights into the molecular mechanisms underlying NSCLC cell proliferation and apoptosis by identifying a new pathway. By targeting SOCS6, miR-1260b mediated by YY1, regulates p38 and ERK expression involved in KIT signaling. A model summarizing these concepts is shown in Fig. 7.

miR-1260b was first discovered to be expressed in vaginal secretions^17^. Analyses of GEO data indicated high expression of miR-1260b in the plasma of NSCLC patients, which was confirmed by our work. Interestingly, miRNAs were not only body fluid specific but also tissue specific. Through human miRNA array, Xu et al.^18^ found overexpression of miR-1260b in NSCLC was associated with cell invasion and metastasis. Here, we observed that miR-1260b level was elevated in NSCLC tissues. In line with our verification, miR-1260b was also increased in prostate cancer^19^, colorectal cancer^20^, and hepatocellular carcinoma^21^. miR-1260b could enhance cell proliferation in renal cell carcinoma^22^. Consistent with this result, abnormal expression of miR-1260b was obviously correlated with tumor size in our study. In vitro, NSCLC cell lines had higher miR-1260b expression than 16HBE cell. ISH demonstrated that miR-1260b was expressed primarily in malignant epithelial cells. Therefore, we can infer that miR-1260b functions as an important molecular in NSCLC progression. Subsequently, the vitro experiments exhibited that upregulated miR-1260b in SPCA1 cells exerted a promotion effect on cell proliferation and cell cycle, an inhibition effect on cell apoptosis and cell senescence; meanwhile, low expression of miR-1260b in H1299 cells exerted the opposite observation. Analysis of western blot showed upregulation of miR-1260b markedly increased Cyclin D1, Ki67, antiapoptotic protein Bcl-2 and decreased cleaved Caspase-3, p21. Knockdown of miR-1260b markedly decreased Cyclin D1, Ki67, Bcl-2, and increased cleaved Caspase-3, p21. In vivo, we also found that miR-1260b promoted xenograft tumor formation. In summary, we demonstrated that miR-1260b functioned as an oncogene by promoting cell proliferation and inhibiting cell apoptosis in NSCLC.

As well known, miRNAs participate in the development of various diseases by regulating the target genes^23,24^. Here, we identified that SOCS6 was the direct target of miR-1260b. SOCS6, located at a locus (18q22.2), is frequently involved in distinctive cancers by occurring allelic loss of this locus^25^. SOCS6 not only regulates cytosolic or membrane-bound proteins, but also resides in the nucleus and influences the function of nuclear proteins. SOCS6 is regarded as a tumor suppressor gene that inducing apoptosis via modulating the mitochondrial proteins^26^. SOCS6 also can suppress gastric cell growth and colony formation by eliciting intrinsic apoptotic pathways^27^. In our work, SOCS6 was significantly upregulated in tumor tissues, and was inversely correlated to miR-1260b. Through performing a rescue experiment, we verified that reconstitution or knockdown of SOCS6 partially rescued the miR-1260b-mediated effects. Otherwise, SOCS6 is a positive regulator or negative regulator of many signaling pathways, such as KIT^14^, insulin receptor^28^, PI3K^29^, and FLT3^30^ signaling. KIT (also named C-KIT) is an RTK expressed in specialized goblet cells, and is association with the stem cell niche in the murine colon crypt base^31^. The influence of C-KIT signaling on the enhancement of cell proliferation and apoptosis, along with the aberrant activation frequency of this pathway, prompt us to explore the efficient approaches for repressing it. C-KIT mutations participate in the distinctive diseases including gastrointestinal stromal cancers, acute myeloid leukemia, mast cell leukemia, and melanoma^32^. Here, KIT expression was elevated in response to miR-1260b overexpression and SOCS6 knockdown. Moreover, miR-1260b and si-SOCS6 both enhanced the phosphorylation of p38 and ERK, indicated that the enhanced activity of KIT regulated pathways in response to loss of SOCS6 might be a mechanism by which miR-1260b promotes cell proliferation.

In addition, the upstream regulators such as transcription factors may be responsible for dysregulated expression of miRNAs in cancers. By using JASPAR database, we identified YY1 as an upstream regulator of miR-1260b expression. The transcription regulator YY1 is an important factor of GLI-Kruppel class of zinc finger proteins and involves in multiple biological processes such as cell proliferation, apoptosis, and differentiation^33,34^. As named, YY1 is able to modulate the downstream genes and acts as a master that regulating the epigenetic network^33^. YY1 is increased in many malignant diseases, such as prostate cancer^35^, ovarian cancer^36^, and breast cancer^37^. In addition, recent studies have found that YY1 participates into the biological processes by silencing the expression of a series of miRNAs such as miR-1, miR-29, miR-133, miR-206, and miR-372^38–40^. Thus, YY1 can regulate expression of both protein-coding genes and noncoding genes such as miRNAs in human cancer cells. In our work, ChIP assay indicated the YY1 binds to the binding site upstream of miR-1260b. Downregulation of YY1 led to decreased miR-1260b expression and increased SOCS6 expression, which demonstrates that there is an axis among YY1/miR-1260b/SOCS6 signaling. Downregulated YY1 caused in an inhibition effect of cell proliferation and an induction effect of cell apoptosis, suggesting that YY1 contributed to the proliferation promotion and apoptosis inhibition by modulating miR-1260b transcription.

Our interest in miR-1260b arose from an earlier finding we performed through microRNA array. We found that compared with the serum of healthy normal people, exosomal-miR-1260b was increased in the preoperative serum but reduced in the postoperative serum of patients with NSCLC (data not shown), which leads us to speculate that its potential as a biomarker was a consequence of its release into the blood from tumor cells. However, first this work sought to explore the role of miR-1260b in cell proliferation and apoptosis. Of note, we demonstrated that: (1) an association between NSCLC progression and high or low levels of miR-1260b and SOCS6, respectively; (2) an inverse relationship between miR-1260b and SOCS6; (3) a positive association between miR-1260b and signaling pathways regulated by YY1. The biological properties of tumors depend not only on the tumor cells themselves, but also on the microenvironment of the tumor. Thus, the regulatory mechanisms of miR-1260b involved in NSCLC progression through the tumor microenvironment (such as exosomes) require substantial additional exploration and validation.

In summary, our study has identified a new onco-miRNA, miR-1260b, mediated by YY1, activates KIT signaling by targeting SOCS6 to regulate cell proliferation and apoptosis in NSCLC. These findings have implications in terms of utilizing miR-1260b as a biomarker and warrant further investigation into potential targeting of this miRNA to suppress NSCLC progression.

## Materials and methods

### Clinical samples

In current study, we recruited a total of 120 NSCLC patients, and all of them underwent the thoracoscopic resection in the First Affiliated Hospital of Nanjing Medical University during March 2012 and October 2017. NSCLC tissues and paired adjacent specimens were preserved in liquid nitrogen after resection until use. All plasma samples (*n* = 90) were collected from NSCLC patients between May 2016 and December 2017 in our hospital. Age-matched healthy human plasma samples (*n* = 30) were selected from the Physical Examination Center in our hospital. And every patient had written informed consent. This research was approved by the department of Ethics Committee of our hospital. The clinical characteristics of all the patients involved in current study were classified based on World Health Organization classification.

### Cell culture

A total of five human NSCLC cell lines (SPCA1, A549, H358, SK-MES-1, and H1299) and one human bronchial epithelioid cell line (16HBE) was involved in current study. All the cell lines were bought from the American Type Culture Collection. Cells in this study were cultured in RPMI-1640 medium + 10% fetal bovine serum along with penicillin (100 U/ml) and streptomycin (100 μg/ml, Invitrogen, Carlsbad, CA) in an incubator containing 5% CO_2_ at 37 °C.

### Cell transfection

Selected NSCLC cell lines SPCA1 and H1299 were transfected with constructed lentiviral vectors (Lv2-pGLV-u6-puro, GenePharma, Shanghai, China). The empty lentiviral vector was taken as a control. Based on the protocols, cells were infected with lentiviruses and screened with puromycin.

Besides, selected cell lines were transfected with SOCS6 siRNA (GenePharma, Shanghai, China), negative control siRNA (GenePharma, Shanghai, China), pcDNA3.1-SOCS6 (GenePharma, Shanghai, China), empty pcDNA3.1 plasmid (GenePharma, Shanghai, China). The siRNA for YY1 was also obtained from GenePharma. For plasmid and siRNA transfections, cells were transfected with Lipofectamine 2000 (Thermo Fisher Scientific, Shanghai, China) based on the protocols.

### MicroRNA ISH

To determine miR-1260b level in human NSCLC tissues and adjacent tissues, the situ hybridization was involved in this study. All paraffin-embedded tissue sections were dewaxed and rehydrated, and were digested using Proteinase K (20 μg/mL) for 30 mins at 37 ℃. After digestion, tissue sections were washed twice with PBS and incubated with hsa-miR-1260b probe premixed solution (8 ng/μl) (Servicebio, Wuhan, China) overnight at 37 ℃. Anti-DIG-HRP was subsequently added for 40 min. All sections were stained by 3, 3-diaminobenzidine (DAB) solution and positive results were exhibited as brownish yellow. The nuclei were counterstained with hematoxylin. Pictures were imaged in a microscope (Nikon, Japan).

### Quantitative real-time PCR analysis

For determination of miR-1260b expression level, the miRNA UPL probe assay was used. By using TRIzol reagent (Invitrogen, USA), total RNA was extracted from the tissues and cells based on the standard instructions.

mRNA levels were analyzed with a SYBR Premix Ex Taq kit (Takara, Japan), PrimeScript™ II Reverse Transcriptase (Takara, Japan) was involved to generate cDNA. GAPDH was used for normalization of qRT-PCR data. All the experiments were performed three times independently.

### CCK-8 assay

Transfected NSCLC cells (6 × 10^3^/ well) were incubated into the plates (96 well) and then were cultured with CCK-8 solution (10 μl, Beyotime, Shanghai, China) for 4 h. By using a spectrophotometer (Thermo Scientific, Rockford, IL, USA), the absorbance was examined at 450 nm (A450).

### EdU analysis

EdU assay was carried out using EdU Apollo567 in Vitro Flow Cytometry Kit (RiboBio, Nanjing, China) based on the instructions. Transfected cells were incubated with EdU (50 μm) for 2 h. Through using a fluorescence microscope the positive cells were screened by Apollo and DAPI staining. The EdU incorporation rate was showed as the ratio of EdU-positive to total DAPI-positive cells (blue cells).

### Flow cytometric assessment

For detection of apoptotic cells, the Annexin V-FITC/PI Apoptosis Detection Kit (Vazyme, Nanjing, China) was involved in current study and performed based on the protocol. For determination of cell cycle, transfected cells in 70% cold ethanol overnight were stained with propidium iodide (PI) (Vazyme, Nanjing, China) for 30 min. The analysis was performed in a BD FACSCanto II (BD Biosciences, USA) flow cytometry and FlowJo software.

### SA-β-gal staining

Based on the manufacturer’s protocol, the Senescence β-Galactosidase Staining Kit (Beyotime, Shanghai, China) was recruited to examine the senescent cells. Transfected cells were planted onto the plates (six-well) and cultured overnight. Then, all the cells were washed with Hanks Balanced Salt Solution (Gibco, NY, USA), and fixed by β-galactosidase staining fixative (1 ml/well) at room temperature for 15 mins. Then, the premixed β-gal staining solution (1 ml/well) was added. After staining, all the cells were imaged by a microscope (Nikon, Japan).

### Luciferase report assay

The 3′-UTR sequence or the mutant sequence of SOCS6 was cloned into pGL3 promoter vector (Genscript, Nanjing, China). SPCA1 cells were transfected with miR-1260b mimic or negative control. After transfection, cells were transfected with the pGL3–SOCS6 3′-UTR constructs (0.12 μg) by Lipofectamine 2000 reagent (Invitrogen, USA). The activity was evaluated with Luciferase Assay System (Promega, USA). The Renilla activity was taken as a normalization of luciferase activity.

### Western blotting

Total protein of cells was isolated with RIPA buffer (Beyotime, Shanghai, China). The protein lysates were separated by SDS-PAGE and then transferred to membrane. Primary antibodies used in Western blotting were Cyclin D1 (Cell Signaling Technology, 2978), Bcl-2 (Cell Signaling Technology, 4223), Ki67 (Abcam, ab92742), Caspase-3 (Cell Signaling Technology, 9665), p21 (Cell Signaling Technology, 8831), SOCS6 (Abcam, ab197335), KIT (Cell Signaling Technology, 3392), p38 (Cell Signaling Technology, 14451), phospho-p38 (Cell Signaling Technology, 4511), ERK (Cell Signaling Technology, 9102), phospho-ERK (Cell Signaling Technology, 8544), GAPDH (Cell Signaling Technology, 5174).

### In vivo xenograft model

In vivo xenograft experiments were approved by Animal Care and Use Committee of Nanjing Medical University. Twelve male NOD/SCID mice (8-week old) were purchased from the Animal Center of Nanjing Medical University, and then were randomly divided into four groups (*n* = 3 per group). SCPA1 cells stably upregulating miR-1260b and H1299 cells stably downregulating miR-1260b were, respectively, injected subcutaneously into both flanks of mice. Tumor size was monitored every 5 days. Five weeks later after injection, the mice were killed. The volume of all the samples was calculated using the formula: tumor volume = volume = (width^2^ × length)/2.

### Immunohistochemistry (IHC)

All the extracted samples were fixed with formalin (4%) and embedded in paraffin. Mice tumor tissues were sliced into tissue sections (5 μm thickness) and incubated with antibodies for SOCS6 (Abcam, ab197335) and Ki67 (Abcam, ab92742) overnight at 4 ℃. The sections were incubated with the secondary HRP Goat Anti-Rabbit IgG (Abcam, ab205718) at room temperature for 1 h, and then were stained with DAB solution. The nuclei were counterstained with hematoxylin. An Olympus microscope (Olympus, Tokyo, Japan) was used to evaluate the images.

### TUNEL technology

For detection and quantification of apoptosis at single cell level, we recruited the In Situ Cell Death Detection Kit, POD (Sigma-Aldrich, Shanghai, China). Based on the protocol, tissue sections were dewaxed and rehydrated, and then were incubated for 20 min with Proteinase K working solution. Tissue sections were subsequently treated by Citrate buffer (200 ml, 0.1 m, pH6.0) and were applied by microwave irradiation for 1 min. After tissue sections were washed by PBS for twice, the slides were immersed in Tris-HCl (0.1 m, pH7.5) containing 3% bovine serum albumin and 20% normal bovine serum at room temperature for 30 mins. Then, all slides were incubated with TUNEL reaction mixture for 60 min in a humidified atmosphere in the dark. All sections were taken pictures by using a microscope (Nikon, Japan).

### Chromatin immunoprecipitation (ChIP)

ChIP analysis was conducted using ChIP assay kit (Millipore, 17-610) based on the manufacturer’s instructions. A total of 1 × 10^7^–5 × 10^7^ cells were collected and 1% formaldehyde (Bio-Rad, CA, USA) was used to cross-link the proteins to the DNA for 25 min. Chromatin was sheared to fragments with size of 100–500 bp by sonicating the lysate. After dislodged insoluble substance by centrifugation, 100 μl DNA/protein complexes were taken as input. The samples were incubated with YY1 antibody (Cell Signaling Technology, 63227), normal rabbit IgG antibodies (Cell Signaling Technology, 2729), and protein A/G beads overnight at 4 ℃. After incubation at 65 ℃ for 4 h, the crosslinking of input and the samples were reversed. Then, phenol/chloroform (Invitrogen) was involved to recover DNA from the samples. Promoter binding was evaluated via PCR with primers of miR-1260b upstream region.

### Statistical analysis

All data in current study were showed as mean ± standard deviation. *χ*^2^ test was involved for analysis of clinicopathological data. Pearson correlation analysis was used to assess the correlation of miR-1260b and SOCS6. Student’s *t* test was used to analysis the significant differences. *P* < 0.05 was regarded as significant. In this study, the data were analyzed using STAT11 and GraphPad Prism (version 5.01; GraphPad Software, Inc, La Jolla, CA) statistical software.

## Supplementary information

Additional file 1: Figure. S1

Additional file 2: Figure. S2

Additional file 3: Figure. S3

Additional file 4: Figure. S4

Additional file 5: Figure. S5

Additional file 6: Figure. S6

supplemental figure legends
