# Correction: miR-1260b, mediated by YY1, activates KIT signaling by targeting SOCS6 to regulate cell proliferation and apoptosis in NSCLC

**DOI:** 10.1038/s41419-020-2452-x

**Published:** 2020-04-21

**Authors:** Yang Xia, Ke Wei, Feng-Ming Yang, Liu-Qing Hu, Chun-Feng Pan, Xiang-Long Pan, Wei-Bing Wu, Jun Wang, Wei Wen, Zhi-Cheng He, Jing Xu, Xin-Feng Xu, Quan Zhu, Liang Chen

**Affiliations:** 1 Department of Thoracic Surgery, The First Affiliated Hospital of Nanjing Medical University, Nanjing, 210029 China; 2 Department of Oncology, The First Affiliated Hospital of Nanjing Medical University, Nanjing, 210029 China; 3 Department of Anesthesiology, The First Affiliated Hospital of Nanjing Medical University, Nanjing, 210029 China

**Keywords:** Non-small-cell lung cancer, miRNAs, Mechanisms of disease

Correction to: *Cell Death and Disease*

10.1038/s41419-019-1390-y published online 8 February 2019

Since online publication of this article, the authors noticed that there was an error in the labeling of Figs. 3b and 6k. In Fig. 3b, the sequence of SOCS6 3’UTR Mutant was incorrect. In Fig. 6k, the left and right group labels for the cell apoptotic rates histogram were labeled inversely. The corrected figures are provided below, and both the PDF and HTML versions of the article have been updated.

**Figure:**
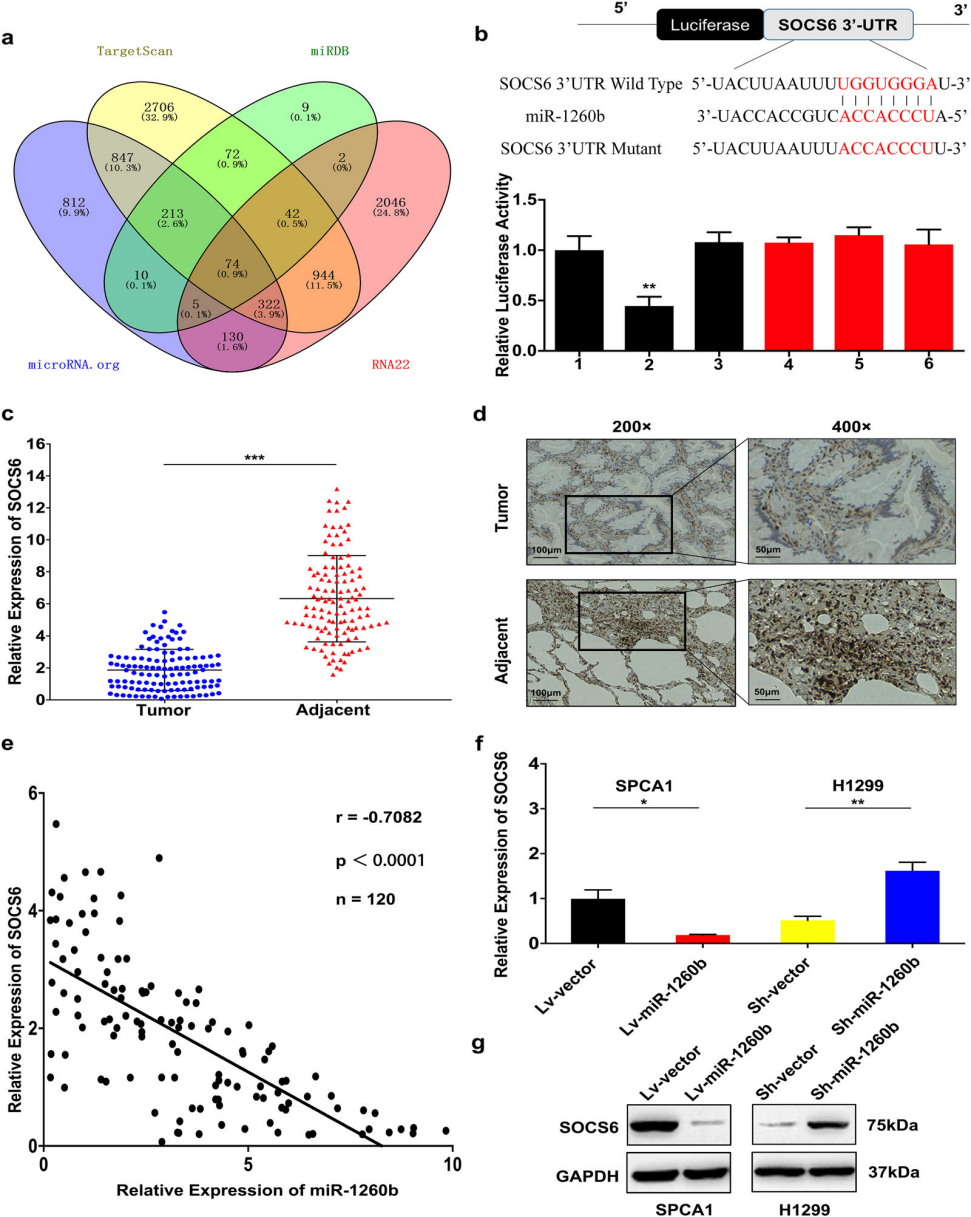
**Fig. 3**

**Figure:**
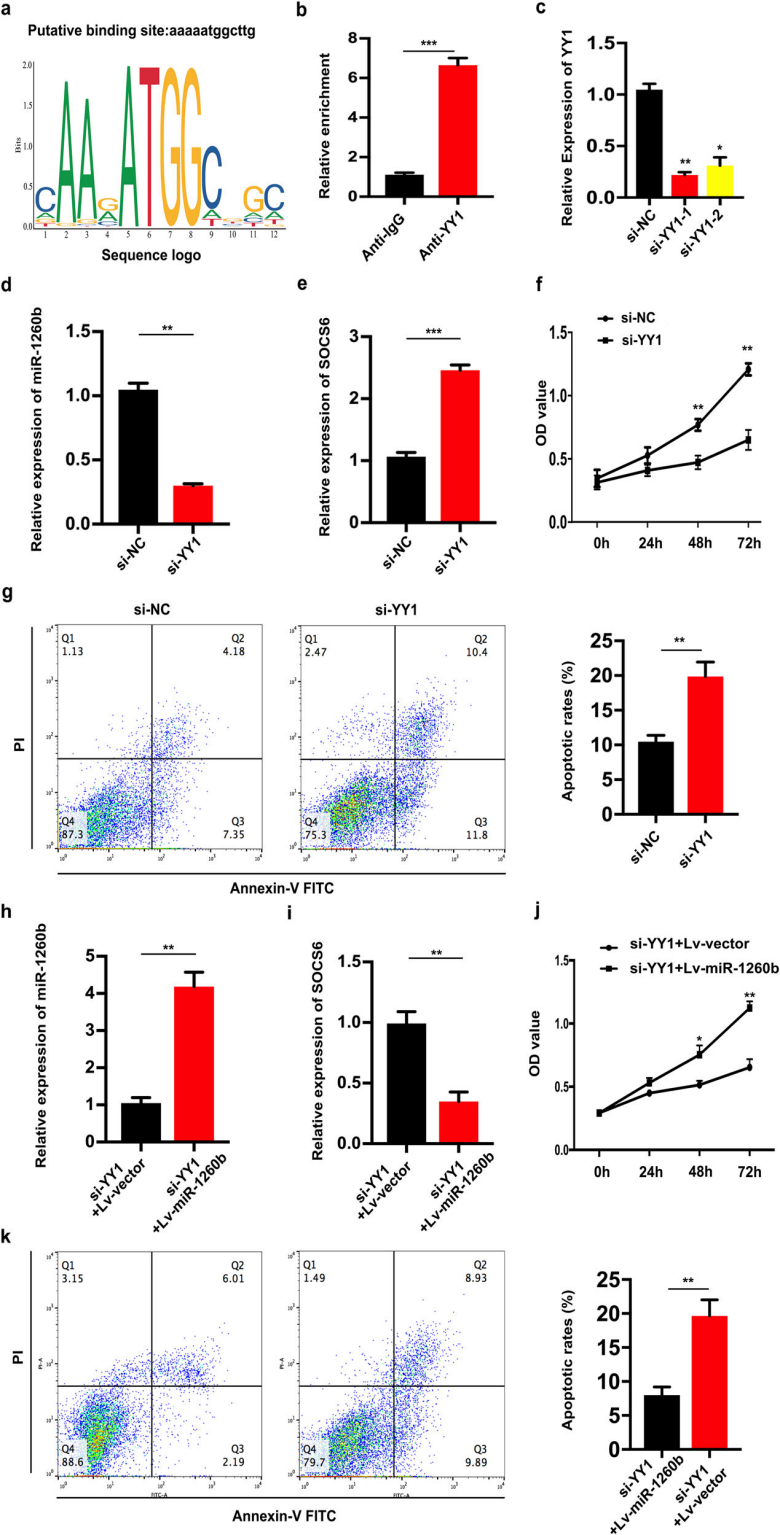
**Fig. 6**

The authors confirm that these errors do not affect the results and conclusions of the study. The authors apologize for any inconvenience caused.

