# Correction: miR-1260b, mediated by YY1, activates KIT signaling by targeting SOCS6 to regulate cell proliferation and apoptosis in NSCLC

**DOI:** 10.1038/s41419-026-08782-2

**Published:** 2026-05-11

**Authors:** Yang Xia, Ke Wei, Feng-Ming Yang, Liu-Qing Hu, Chun-Feng Pan, Xiang-Long Pan, Wei-Bing Wu, Jun Wang, Wei Wen, Zhi-Cheng He, Jing Xu, Xin-Feng Xu, Quan Zhu, Liang Chen

**Affiliations:** 1 Department of Thoracic Surgery, the First Affiliated Hospital of Nanjing Medical University, Nanjing, 210029 China; 2 Department of Oncology, the First Affiliated Hospital of Nanjing Medical University, Nanjing, 210029 China; 3 Department of Anesthesiology, the First Affiliated Hospital of Nanjing Medical University, Nanjing, 210029 China

Correction to: *Cell Death & Disease* 10.1038/s41419-019-1390-y, published online 08 February 2019

Since online publication of this article, the authors noticed that there was a mistake “Figure S6c-4 was inadvertently duplicated in Figure 2d-3”. This mistake occurred due to a mislabeling of image files during preparation. The corrected figure is provided below. The authors confirm that this mistake does not affect the results and conclusions of the study. The authors sincerely apologize for the mistake and any inconvenience caused.

**Amended Figure S6** 
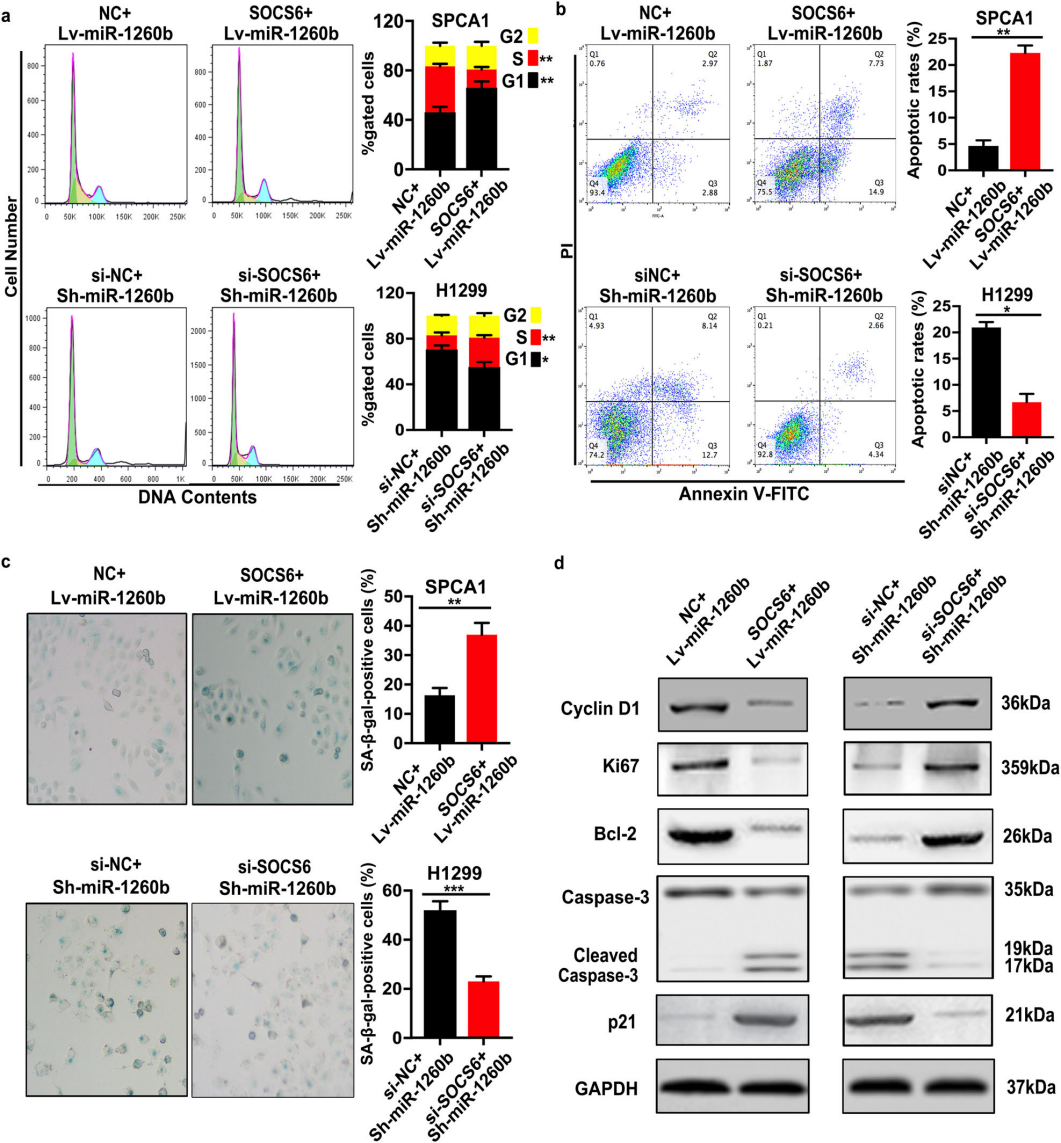


**Original data of Figure S6c-1** 
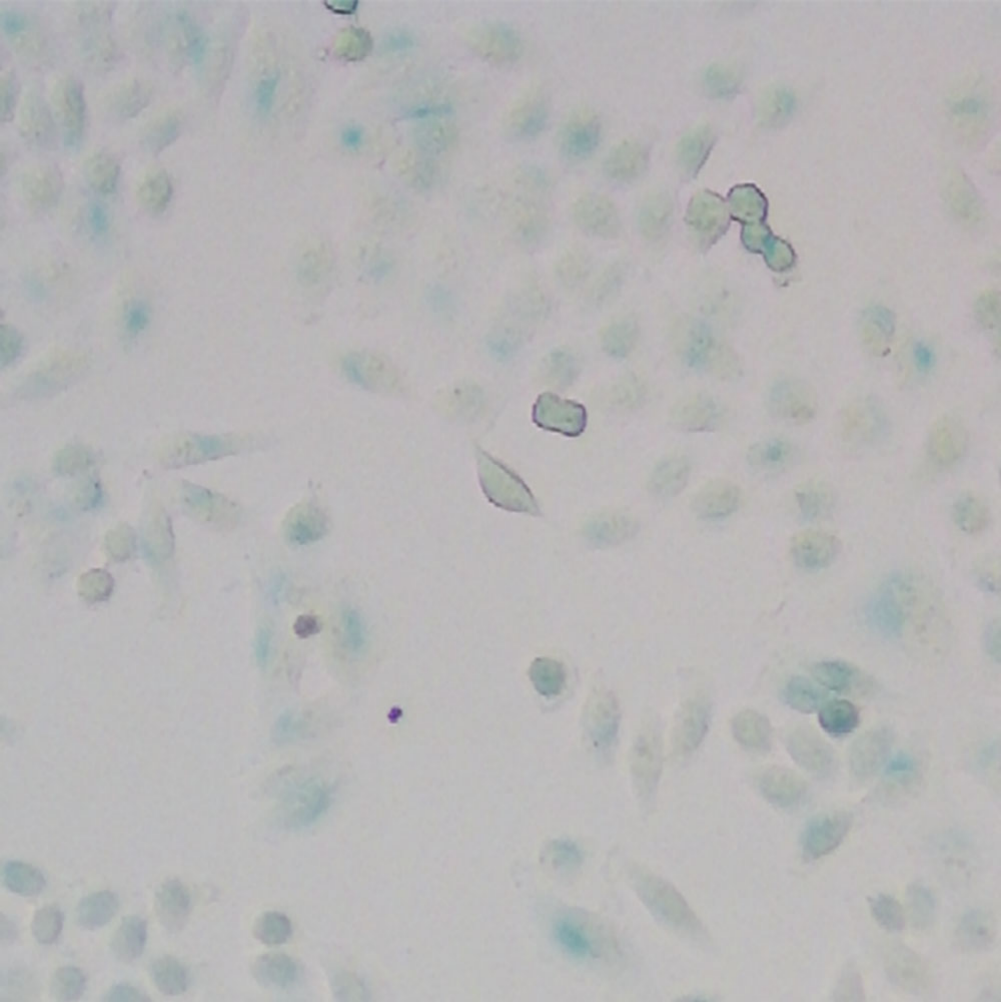


**Original data of Figure S6c-2** 
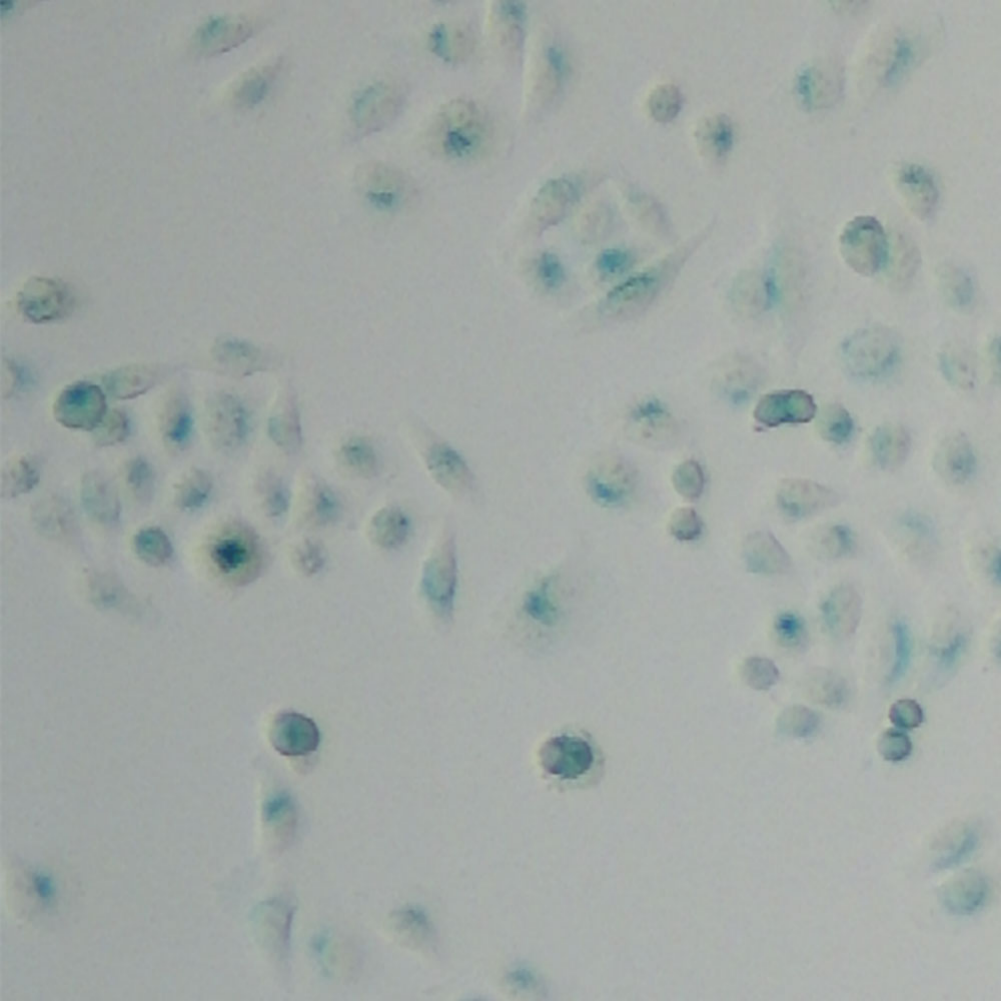


**Original data of Figure S6c-3** 
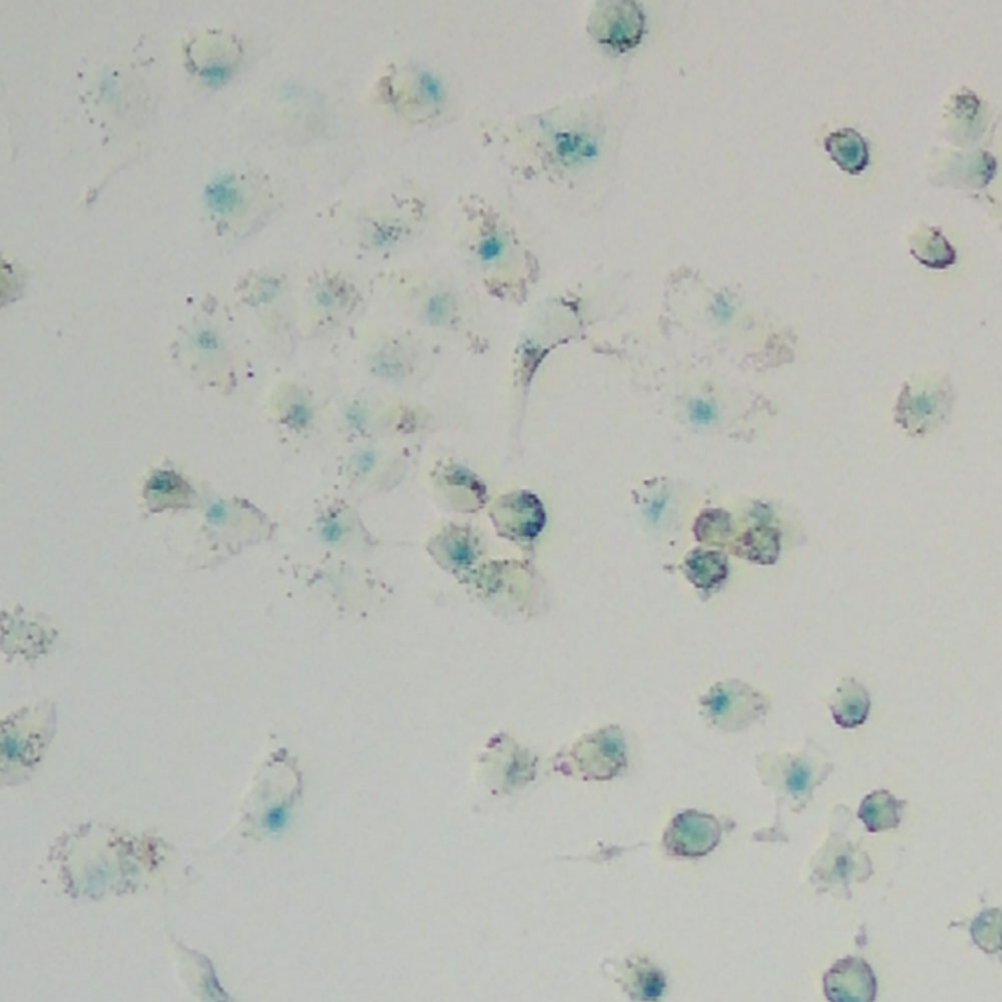


## Supplementary information

Amended Figure S6

