# Supplementary figures and images for: miR-1260b, mediated by YY1, activates KIT signaling by targeting SOCS6 to regulate cell proliferation and apoptosis in NSCLC
Source: Cell Death Dis. 2019 Feb 8;10(2):112. doi: 10.1038/s41419-019-1390-y (PMC6368632; doi:10.1038/s41419-019-1390-y)

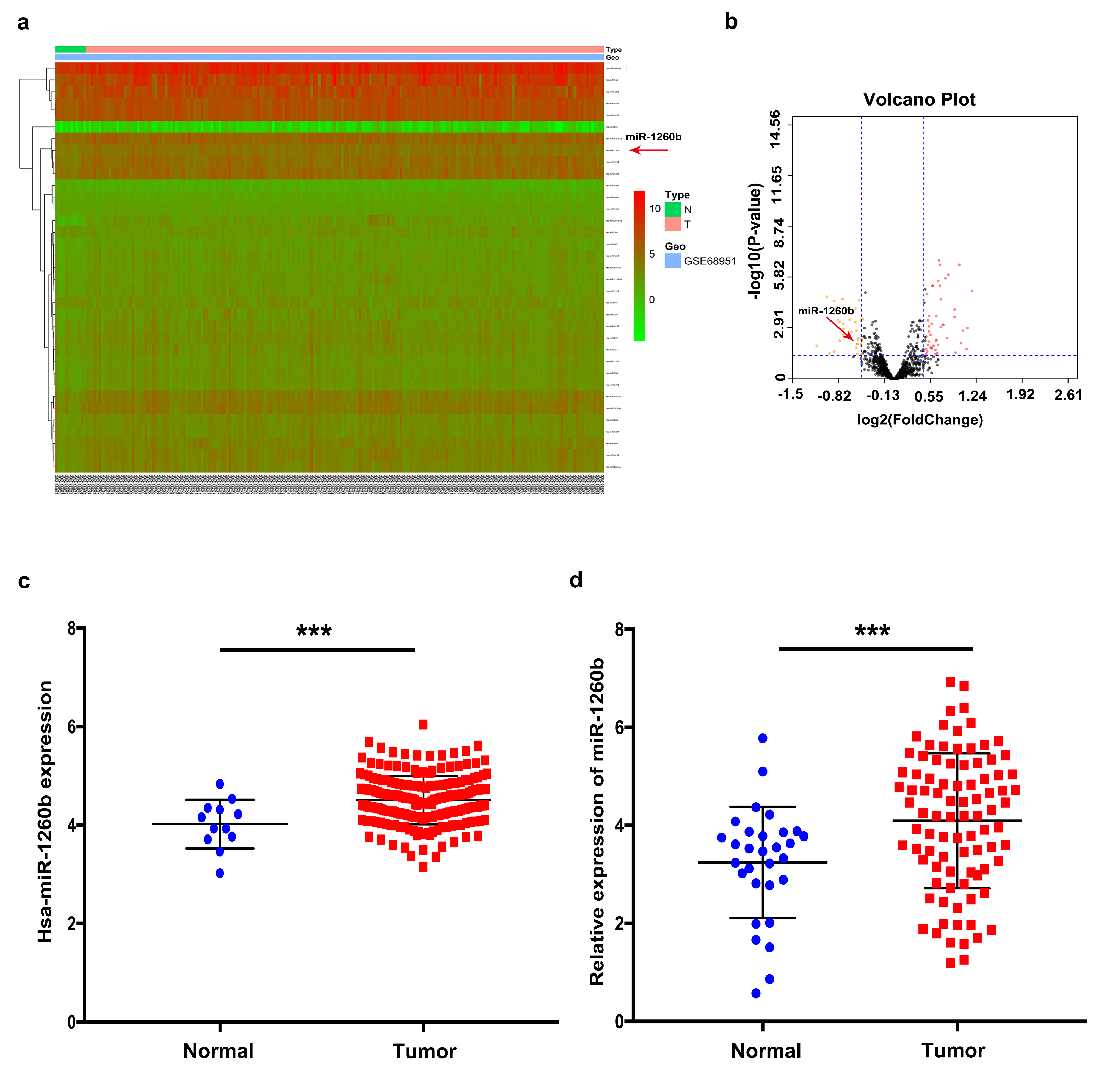

Supplement: Supplementary file 1 — Additional file 1: Figure. S1 [file 41419_2019_1390_MOESM1_ESM.tif]

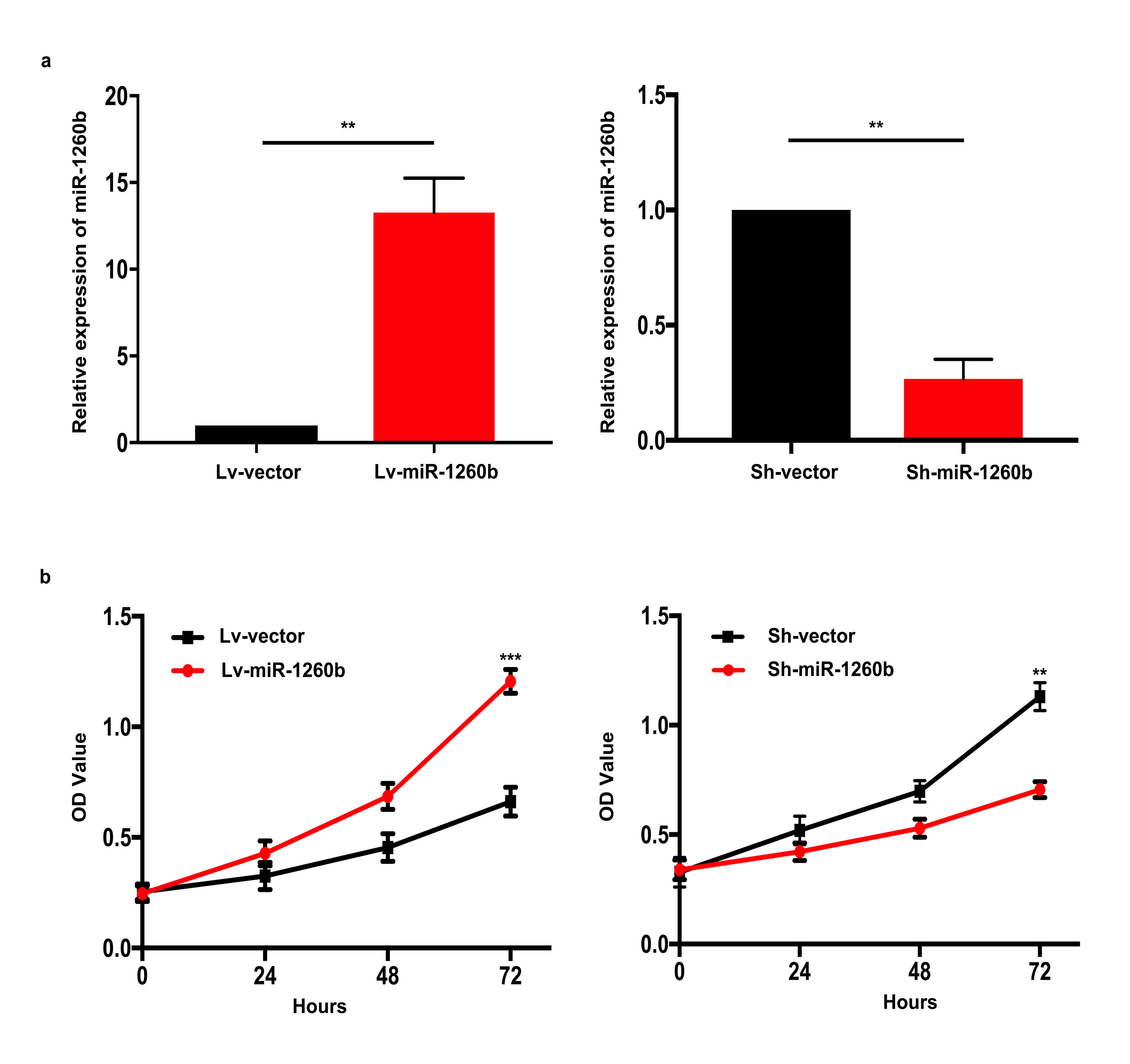

Supplement: Supplementary file 2 — Additional file 2: Figure. S2 [file 41419_2019_1390_MOESM2_ESM.tif]

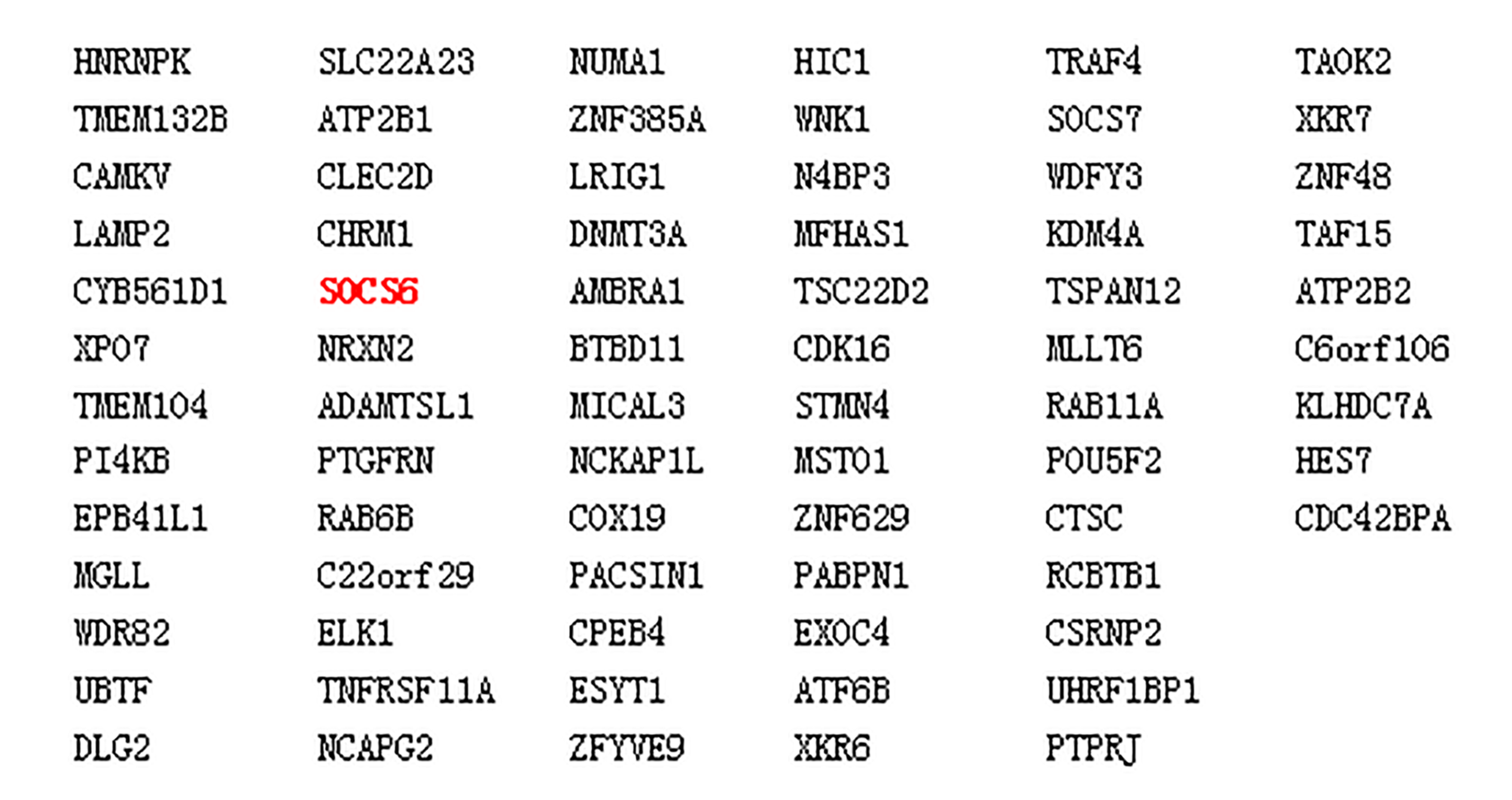

Supplement: Supplementary file 3 — Additional file 3: Figure. S3 [file 41419_2019_1390_MOESM3_ESM.tif]

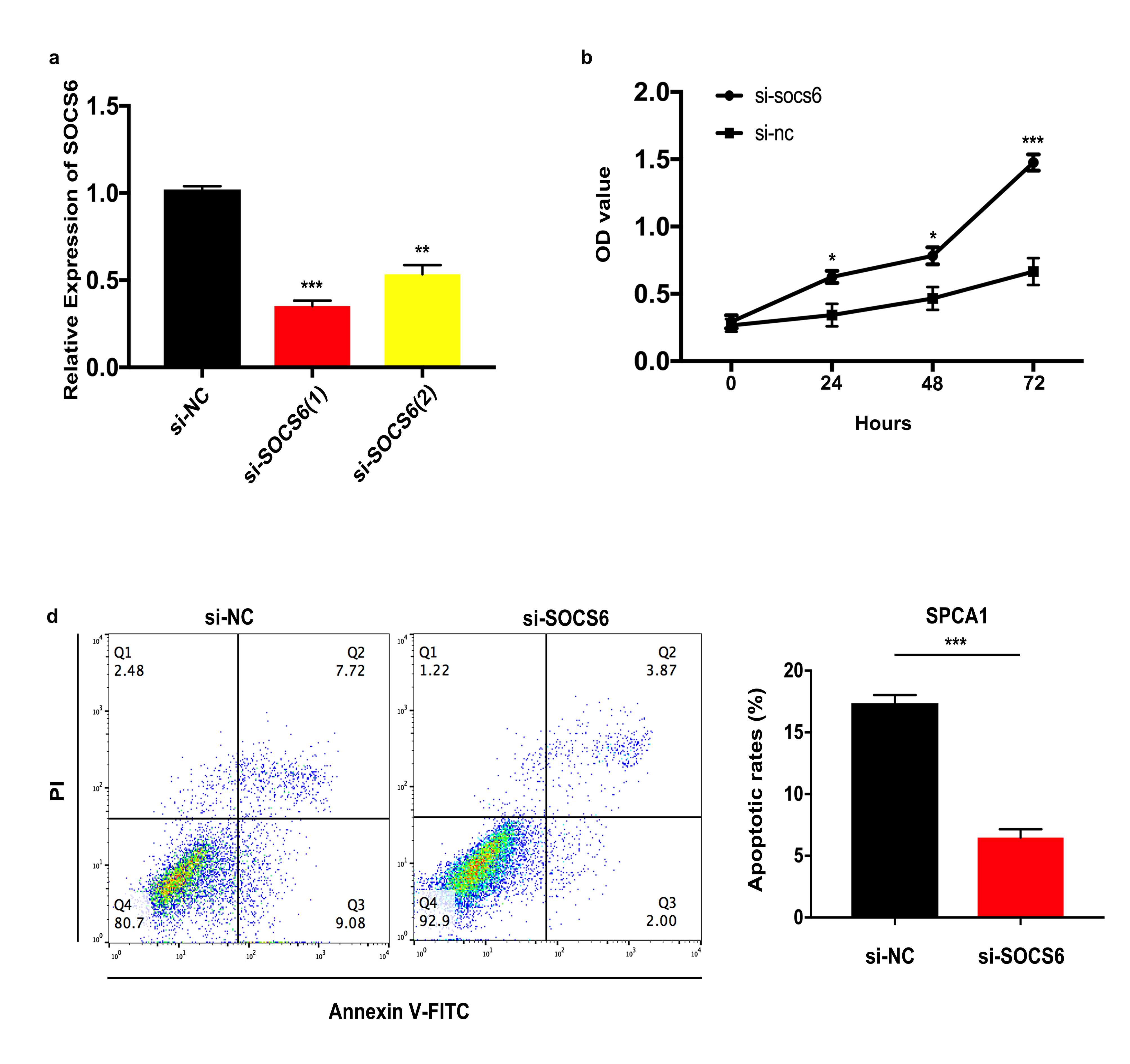

Supplement: Supplementary file 4 — Additional file 4: Figure. S4 [file 41419_2019_1390_MOESM4_ESM.tif]

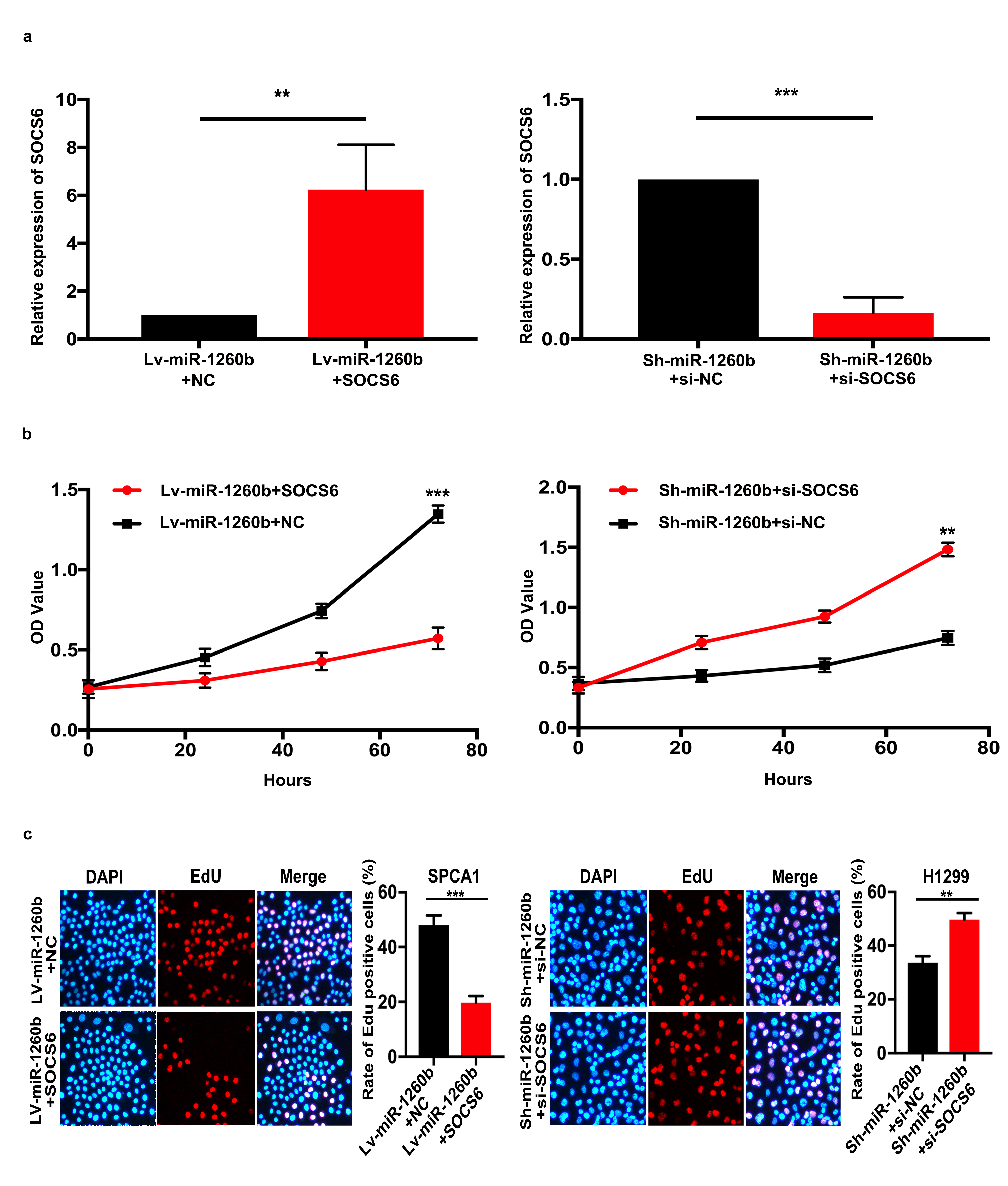

Supplement: Supplementary file 5 — Additional file 5: Figure. S5 [file 41419_2019_1390_MOESM5_ESM.tif]

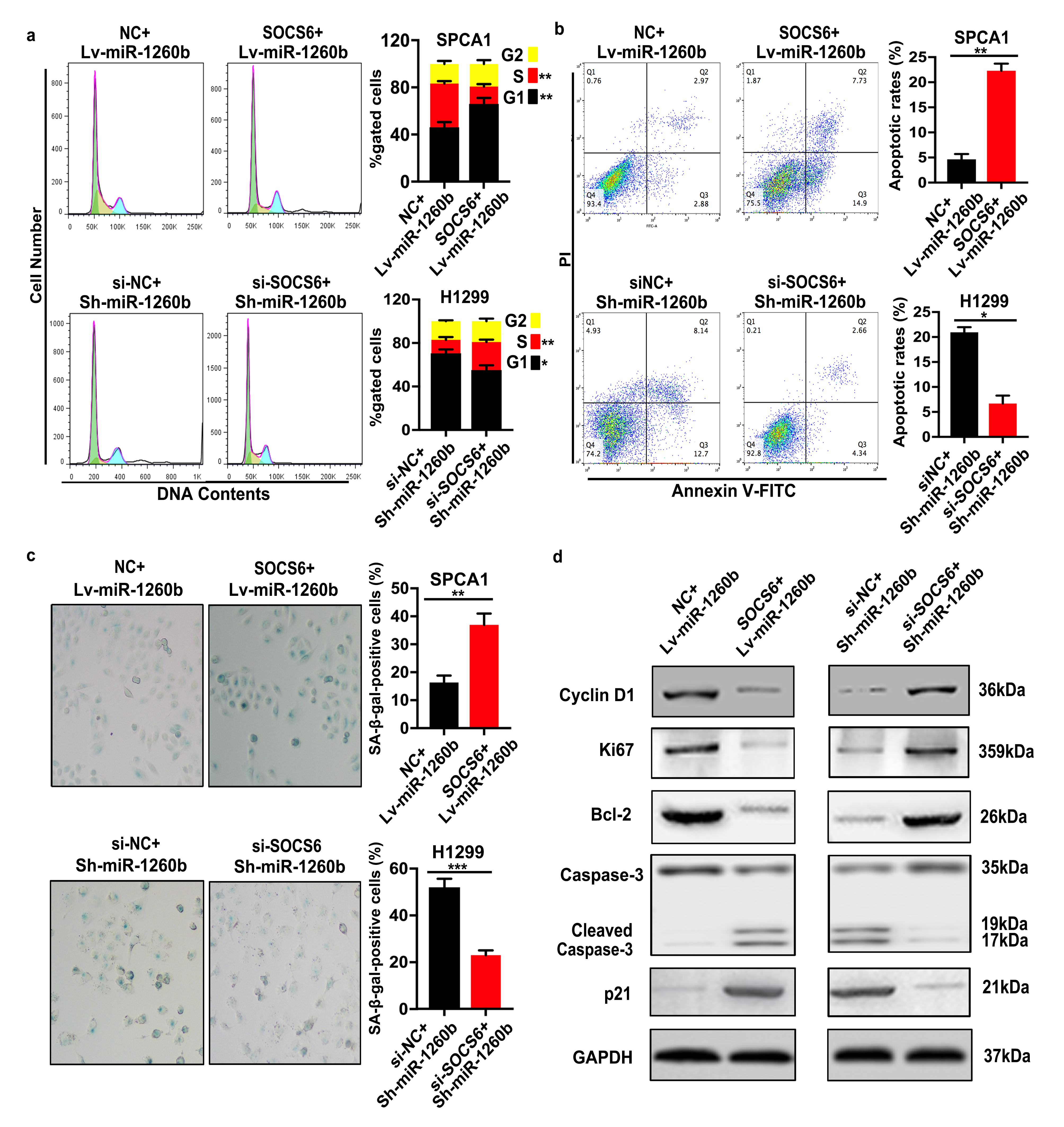

Supplement: Supplementary file 6 — Additional file 6: Figure. S6 [file 41419_2019_1390_MOESM6_ESM.tif]
