# Supplementary material for: miR-1260b, mediated by YY1, activates KIT signaling by targeting SOCS6 to regulate cell proliferation and apoptosis in NSCLC
Source: Cell Death Dis. 2019 Feb 8;10(2):112. doi: 10.1038/s41419-019-1390-y (PMC6368632; doi:10.1038/s41419-019-1390-y)
Supplement: Supplementary file 7 — supplemental figure legends [file 41419_2019_1390_MOESM7_ESM.docx]

**Figure S1. miR-1260b was increased in the plasma of NSCLC patients.** **a, b** The data of miRNAs expression in plasma of NSCLC patients were downloaded from GEO database and exhibited as a heatmap or a Volcano Plot. **c** The level of miR-1260b in plasma of NSCLC patients and normal cohort was found in GEO database. **d** qRT-PCR was used to examine the relative expression of miR-1260b in plasma specimens of both NSCLC patients (n=90) and age-matched healthy human plasma samples (n=30). U6 was used as an internal control. The data expressed as the mean ± SD (**P*<0.05; ***P*<0.01; ****P*<0.001).

**Figure S2. Gain- or loss-function of miR-1260b regulated cell proliferation.** **a** The transfection efficiency of lentivirus vector transfection was detected by qRT-PCR. **b** To determine the role of miR-1260b in proliferation of NSCLC cell lines, we recruited CCK8 assay. The data expressed as the mean ± SD (**P*<0.05; ***P*<0.01; ****P*<0.001).

**Figure S3. The resultant list of 74 genes**

**Figure S4. SOCS6 knockdown promoted cell proliferation and inhibited apoptosis. a** Cell proliferation was verified by CCK-8 assay in siRNA transfected cells. **b** Down-expression of SOCS6 suppressed cell apoptosis in flow cytometric analysis. The data expressed as the mean ± SD (**P*<0.05; ***P*<0.01; ****P*<0.001).

**Figure S5. Upregulation or downregulation of SOCS6 partially rescued the effect of miR-1260b on cell proliferation. a** Transfection efficiency of co-transfected cell lines was determined by qRT-PCR. **b** Cell proliferation was evaluated through CCK-8. **c** To further determine cell proliferation, EdU assay was performed. The data expressed as the mean ± SD (**P*<0.05; ***P*<0.01; ****P*<0.001).

**Figure S6. Reconstitution or knockdown of SOCS6 partially rescued the miR-1260b-mediated effects on cell cycle and apoptosis.** **a** Flow cytometric analysis, the effect of miR-1260b on cell cycle was abolished after co-transfected with SOCS6 plasmid or siRNA. **b** The inhibition of cell apoptosis in miR-1260b up-regulated cells was cancelled by SOCS6 over-expression. **c** The effect of miR-1260b on cell senescence was reversed after co-transfected with SOCS6 plasmid or siRNA. **d** The expression levels of Cyclin-D1, Ki67, Bcl-2, Caspase-3, p21 were determined by western blotting in co-transfected cells. GAPDH was used as an internal control. The data expressed as the mean ± SD (**P*<0.05; ***P*<0.01; ****P*<0.001).
