# Supplementary figures and images for: Correction: miR-1260b, mediated by YY1, activates KIT signaling by targeting SOCS6 to regulate cell proliferation and apoptosis in NSCLC
Source: Cell Death Dis. 2026 May 11;17(1):438. doi: 10.1038/s41419-026-08782-2 (PMC13161324; doi:10.1038/s41419-026-08782-2)

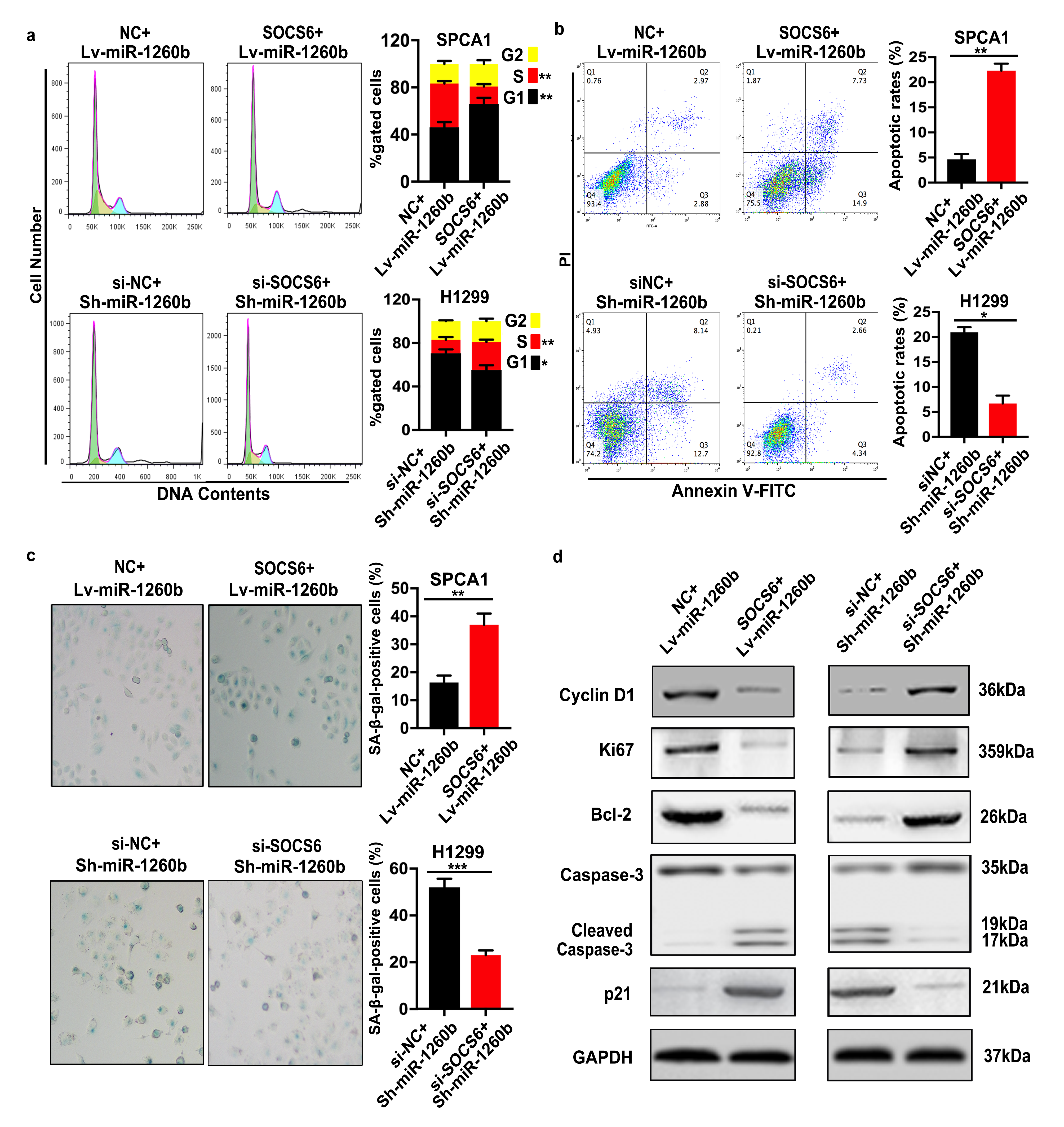

Supplement: Supplementary file 1 — Amended Figure S6 [file 41419_2026_8782_MOESM1_ESM.tif]
